# NF-κB-dependent and independent inflammatory responses during acute HIV-1 infection in a model of human microglia

**DOI:** 10.3389/fimmu.2026.1912292

**Published:** 2026-08-14

**Authors:** Aracelly Gaete-Argel, Catarina Ananías-Sáez, Cecilia Rojas-Fuentes, Sebastian Giraldo-Ocampo, Daniela Jara, Tomás Hernández-Díaz, Camila Ortega-Orellana, Delia López-Palma, Hisashi Akiyama, Suryaram Gummuluru, Pablo A. González, Fernando Valiente-Echeverría, Ricardo Soto-Rifo

**Affiliations:** 1 Laboratorio de Virología Molecular y Celular, Núcleo Interdisciplinario de Microbiología, Instituto de Ciencias Biomédicas (ICBM), Facultad de Medicina, Universidad de Chile, Santiago, Chile; 2 Center for HIV/AIDS Integral Research (CHAIR), Facultad de Medicina, Universidad de Chile, Santiago, Chile; 3 Instituto Milenio en Inmunología e Inmunoterapia, Santiago, Chile; 4 Department of Virology, Immunology & Microbiology, Boston University Chobanian & Avedisian School of Medicine, Boston, MA, United States; 5 Facultad de Ciencias Biológicas, Pontificia Universidad Católica de Chile, Santiago, Chile

**Keywords:** HIV-1, MAVS, microglia, Nef, neuroinflammation, NF-κB

## Abstract

**Introduction:**

Microglia are the main targets of HIV-1 infection in the central nervous system (CNS) and are considered important contributors to chronic neuroinflammation in people living with HIV (PLWH). In this study, we investigated the mechanisms leading to inflammatory responses during acute HIV-1 infection in an adult human microglia model.

**Methods:**

We used an adult human microglia model to characterize inflammatory responses induced by acute HIV-1 infection and analyzed publicly available datasets to assess the relationship between viral loads and cytokine levels in the cerebrospinal fluid of PLWH.

**Results:**

HIV-1 infection induced a subset of pro-inflammatory mediators, including the neurotoxic cytokines IL-6, IL-8, and IL-11, through distinct mechanisms. IL-6 and IL-8 induction depended on activation of the NF-κB signaling pathway mediated by the viral accessory protein Nef, whereas IL-11 expression relied on cellular sensing of Rev-dependent transcripts and subsequent activation of MAVS signaling. Analysis of public datasets revealed a significant correlation between viral loads and IL-6/IL-8 levels in the cerebrospinal fluid of PLWH, suggesting that active HIV-1 replication in microglia contributes to increased CNS pro-inflammatory cytokine levels even under suppressed systemic viral loads.

**Discussion/Conclusion:**

These findings highlight the complexity of inflammatory responses during active HIV-1 replication and provide a mechanistic framework for understanding the contribution of microglia to neuroinflammation in the CNS of PLWH.

## 1 Introduction

HIV-1 infects and persists in the CNS of PLWH despite successful suppression of plasma viral loads due to combined antiretroviral therapy (1–3). Microglia, the sentinels of the CNS responsible for maintaining brain homeostasis, have been shown as the main target for HIV-1 infection (1, 4, 5). Infected microglia are considered major contributors to inflammation in the brain leading to neurologic complications in PLWH (5–9). As such, accumulating evidence from brain tissues (8, 10, 11), human brain organoids (12–14), primary cultures (15), primary-like microglia models (iPSC-derived or monocyte-derived) (14–16), immortalized cell lines (15, 17), and a humanized mouse model (18), have linked HIV-1 infection with the induction of pro-inflammatory responses of microglia. Moreover, studies using recombinant viral proteins indicates that secretion of neurotoxic HIV-1 proteins such as Tat, gp120, Vpr, and Nef may also contribute to neuronal damage in PLWH (19–25). However, while these approaches have provided important insights into HIV-induced neuroinflammation, there are still gaps in our understanding of the molecular mechanisms leading to immune activation of human microglia during active HIV-1 replication. In this context, evidence from monocyte-derived microglia (MDMG) cultures showed that sensing of the HIV-1 intron-containing RNA (icRNA) results in the activation of the MAVS signaling and the secretion of CXCL10 (16). In other myeloid cells, including monocyte-derived macrophages (MDM) and dendritic cells (MDDC), HIV-1 icRNA have been shown to activate the RNA sensor MDA-5 initiating downstream signaling including the IRF5/MAVS axis, which leads to type-I interferon and pro-inflammatory responses (26–29).

Here, we used an adult human microglia cell line to further investigate the mechanisms of HIV-1-driven inflammatory responses during acute viral replication. Our results show that HIV-1 infection results in a transcriptional reprogramming that differs from a classical antiviral response elicited by sensing of non-self RNA. Such a response led to the induction of a subset of cytokines including the neurotoxic pro-inflammatory mediators IL-6, IL-8 and IL-11 through two distinct mechanisms involving the activation of the NF-κB signaling pathway by the viral protein Nef and the induction of MAVS signaling triggered by the sensing of Rev-dependent transcripts by RIG-I-like receptors (RLRs). Interestingly, while this pro-inflammatory response involving IL-6, IL-8 and IL-11 was conserved in other HIV-1 isolates, the closely related human lentivirus HIV-2 triggered the induction of IL-6 and IL-8 but not IL-11, suggesting a specific mechanism of HIV-1 Rev-dependent RNA sensing in this cellular model. Finally, leverage of public data shows a significant correlation between viral loads and IL-6, IL-8, CCL2 and CXCL10 levels but not CCL5 and IL-1β in the cerebrospinal fluids of PLWH even under suppressive cART, providing a link between active replication and increased secretion of pro-inflammatory cytokines in the CNS despite suppressed systemic viral loads.

The data presented here provide novel insights into the complex regulatory networks that mediate pro-inflammatory responses during HIV-1 infection of human microglia providing a mechanistic framework for better understanding neuroinflammation in PLWH.

## 2 Materials and methods

### 2.1 Cell culture

Human cell lines C20 (17) and HEK-293T were maintained in Dulbecco’s Modified Eagle’s Medium (DMEM) (Cat SH30081.02 Hyclone Cytiva) supplemented with 10% FBS (Cat F0926, Sigma), 1% antibiotic-antimycotic (Cat A5955, Sigma) and 1% L-Glutamine (Cat A2916801, Sigma) at 37°C and 5% CO_2_. All cell cultures were routinely tested for mycoplasma following the procedure described in (30).

### 2.2 Reporter cell lines generation

Reporter cell lines used in this study were generated by lentiviral transduction. Briefly, 500,000 HEK-293T cells in 35 mm well plates were transfected with 1.5 μg psPAX2 (Addgene plasmid # 12260), 0.8 μg pCMV VSV-G (obtained from Dr. Phillipe Mangeot) and 1.5 μg reporter lentivector. To generate the p50/p65 NF-κB and the p50/RelB reporter cell lines, lentiviral vectors were pHAGE NFkB-TA-LUC-UBC-GFP-W (Addgene plasmid # 49343) and pLminP_Luc2P_RE38 (Addgene plasmid # 90378), respectively. Media was replaced at 6 hours post-transfection and lentiviral particles were recovered and filtered using 0.45 μm filters 72 hours later. C20 microglia cell lines were transduced with each reporter lentivirus and maintained under normal conditions for 48 hours. For reporter activity assays, reporter cell lines were seeded in triplicate in white-bottom 96 well plates (10,000 cells per well) and infected or treated as indicated. Cells were lysed using 25 μL of Reporter Lysis Buffer (Cat.# E4030, Promega) and incubated at room temperature for 10 minutes in agitation at 500 rpm. Firefly luciferase activity was measured using 25 μL of the Luciferase Assay System substrate (Cat E1501, Promega).

### 2.3 Pseudotyped virus production, quantification and infections

Plasmids used to produce pseudotyped HIV-1 wild type or mutant viruses were previously described in (31). The LAI Infectious Molecular Clone (pLAI.2, ARP-2532) was obtained through BEI Resources, NIAID. The HIV-1 ADA and pRod10 infectious molecular clones were a gift from Theophile Ohlmman´s lab (CIRI, Lyon, France). The pROD10Δenv was generated by digestion of the pROD10 with PmII and ligation of the sequence CCCAGGGTGGGGCAGCCATCACAAC to insert a frameshift and a premature stop codon at the start of the *env* gene. Seven million HEK-293T cells were seeded in a 10 cm dish the day prior to transfection. Cells were co-transfected using 6 μg HIV-1 or HIV-2 molecular clones and 4 μg pCMV VSV-G using Lipofectamine™ 3000 (Cat. #L3000015, ThermoFisher) following manufacturer’s instructions. Media was changed 6 hours post-transfection and reduced to 50% of the initial plate volume. Pseudotyped viruses were collected from the supernatant 72 hours post-transfection, cellular debris was pelleted at 3,000 rpm for 5 minutes at room temperature and the supernatant was diluted 50% in FBS. Viral stocks were aliquoted and stored at -80°C until use. Each viral stock was quantified using the HIV-1 Gag p24 Quantikine ELISA Kit (Cat DHP240B, R&D Systems) following manufacturer’s instructions. To quantify viral copy numbers, an *in vitro*-transcribed RNA standard was used. Briefly, primers containing the SP6 promoter were used to generate a DNA template for the first 4587 nucleotides of the pNL4-3 sequence using the Phusion High-Fidelity DNA Polymerase (Cat F0530S, ThermoFisher) (**Supplementary Table 1**). *In vitro* transcription was performed with the mMessage mMachine SP6 transcription kit (Cat AM1340, ThermoFisher) and the resulting *in vitro* transcribed RNA (IVT RNA) was precipitated and purified according to manufacturer’s instructions (32). Total RNA from 500 μL of each viral stock was extracted with 1 mL TRIzol, followed by addition of 200 μL chloroform and centrifugation at 10,000 rpm for 5 minutes at 4°C. 900 μL of the aqueous phase was recovered and mixed with 500 μL isopropanol (Cat 109634, Merck) and 20 μg of glycogen (Cat R0551, ThermoFisher). After centrifugation at 13,000 rpm for 20 minutes at 4°C, the RNA pellet was washed with 500 μL 70% ethanol and then resuspended in 10 μL ultra-pure water. Ten-fold serial dilutions of the IVT RNA in duplicate and viral RNA samples were amplified by one-step qRT-PCR using the KAPA SYBR^®^ FAST One-Step kit (Cat KK4652, Roche) with specific primers for the HIV-1 US RNA (listed in **Supplementary Table 1**). RNA copies/mL were calculated for each viral stock from the standard curve. Equal copy numbers relative to wild type virus were used in experiments involving the use of mutant viruses, otherwise, 250 ng p24 were used to infect 300,000 cells, followed by media change at 24 hours and incubation until 48 hours post-infection.

Viral stocks of Zika virus (Brazilian isolate BeH819015, GenBank accession n° KX197192) were produced in Vero E6 cells as we described (33). Zika virus stock was diluted in DMEM containing 2% FBS at MOI of 3 and adsorbed for 1 hour at 37°C. After viral adsorption, the media was changed. Infection was led to proceed for 24 hours for analysis.

### 2.4 siRNA transfections, drugs and treatments

Cells were seeded at the desired confluency the day prior transfection and then transfected with siRNA using the Lipofectamine RNAiMAX™ Transfection Reagent (Cat 13778150, ThermoFisher). At 24 hours post-transfection, cells were infected as described above. The following siRNAs were transfected at 10 nM final concentration: RIG-I (sc-61480), MDA-5 (sc-61010), MyD88 (sc-74532) and Control siRNA-A (sc-37007). Silencing of MAVS (sc-75755) was performed at 30 nM with the respective Control siRNA-A (sc-37007). Silencing efficiency was evaluated by western blot using specific antibodies. The NF-κB inhibitor Parthenolide (Cat 70080, Cayman Chemical) was used at 10 μM and added to the cell culture 24 hours post-infection during the media change. DMSO was used as vehicle control. As a positive control for NF-κB induction, cells were treated with 10 ng/mL human recombinant TNF-α (Cat A42551, Invitrogen) for 6 hours before lysis. Ultra-pure water (Cat W3500, Merck) was used as vehicle control. The TLR3 inhibitor CU-CPT 4a (Cat HY-108473, MedChemExpress) was incubated for 24 hours prior to infection at 20 µM. Additional media containing inhibitor was added at 24 hours post-infection during media change. DMSO was used as vehicle control.

### 2.5 RNA extraction and RT-qPCR

Total RNA from cell lysates was extracted by the TRIzol-chloroform method. RNA in the aqueous phase was precipitated with isopropanol (Cat 109634, Merck) by centrifugation at 13,000 rpm at 4°C and washed with 70% ethanol. The RNA pellet was air-dried and resuspended in nuclease-free ultrapure water (Cat W4502, Sigma-Aldrich). Treatment with DNAse was performed following manufacturer’s instructions using the TURBO DNA-free kit (Cat AM 1907, Invitrogen). To generate cDNA, 300 ng total RNA were subjected to retrotranscription using the High-Capacity cDNA Reverse Transcription kit (Cat 4368814, Applied Biosystems). Prior to qPCR, cDNA was 10-fold diluted in ultrapure water and 5 μL were used in each reaction in duplicate. For qPCR analyses, Brilliant II SYBR Green QPCR Master Mix (Cat 600828, Agilent Technologies) or HOT FIREPol^®^ EvaGreen^®^ qPCR Mix Plus (Cat 08-24-00001, Solis Biodyne) were used following manufacturer’s instructions. Primers used in this study are listed in **Supplementary Table 1**. Relative expression normalized to GAPDH mRNA levels was calculated using the x^-ΔΔCt^ method (where x corresponds to the experimentally calculated amplification efficiency of each primer pair).

### 2.6 Bulk RNA-seq

Total RNA from uninfected C20 microglia and microglia infected with a VSVg-pseudotyped HIV-1 NL4.3 virus for 48 hours was extracted as described above. Similarly, total RNA from lipofectamine-treated microglia and microglia transfected with poly(I:C) for 4 hours was also obtained for bulk RNA sequencing. Two (HIV-1) or three (poly(I:C)) independent biological replicates were performed for each condition. RNA quality control, library preparation and RNA sequencing were performed by Genoma Mayor Company (Chile), using Illumina HiSeq2000 technology.

### 2.7 Bioinformatic analysis

For RNA-sequencing data analysis, raw sequencing reads (paired-end FASTQ files) were first subjected to quality control (QC) using FastQC version 0.11.9 to assess per-base sequence quality, GC content, sequence duplication levels, and adapter contamination. As an initial pre-processing step, reads were screened with Bowtie2 version 2.5.1 for removal of contaminant/rRNA-mapping reads. To remove adapter sequences and low-quality bases, reads were trimmed using Trim Galore version 0.6.4 (which leverages Cutadapt). Reads shorter than a minimum length threshold after trimming were discarded, and additional fixed-length trimming with fastq_trimmer of Fastx-toolkit version 0.0.13 was applied when required to standardize read length across libraries. The cleaned reads were then aligned to the Human Reference Genome (GRCh38.p14) using BWA-MEM version 0.7.17: 23, r1188. Gene-level read counts were computed using featureCounts from Rsubread package version 2.14.2 (part of Bioconductor version 3.17) by assigning aligned fragments to annotated genomic features (gene models) to generate a sample-by-gene count matrix. Differential gene expression analysis was performed in R using DESeq2 version 1.40.2, which models raw count data using negative binomial generalized linear models and applies internal normalization (size factors) to account for library depth. Resulting p-values were corrected for multiple testing using the Benjamini–Hochberg procedure, and genes were considered differentially expressed based on an FDR-adjusted p-value threshold (FDR < 0.05) and an effect-size cutoff (log2FC ≥1 or log2FC ≤-1). Functional interpretation of differentially expressed gene lists (up- and down-regulated sets analyzed separately) was conducted by Gene Ontology (GO release 2024) over-representation analysis using Enrichr and GO resources. Transcription factor (TF) enrichment and transcriptional regulatory network analyses were performed using the TRRUST v2 regulatory interaction database (34) with custom analyses implemented in Python 3, overlap-based enrichment with multiple-testing correction. TF–target interactions were imported from TRRUST-derived Excel tables (one per condition). Transcription factors (TFs) were ranked by the enrichment statistic provided in the input, and the top 10 TFs were selected. For each condition, a directed TF→target network was constructed including the selected TFs, all their unique target genes, and edges representing TF–target links. Gene Ontology (GO) functional annotation was derived from a local GO DAG (go-basic.obo) and gene–GO mappings from GOA (*.gaf). GO terms were collapsed into three primary functional classes (Inflammation, Cell activation, Cell growth/survival) using curated anchor term sets for each class; a GO term supported a class if it was an anchor or a descendant of an anchor in the GO DAG. For each gene (target), a class score was computed as the number of its annotated GO terms mapping to each class. Target genes were assigned to the class with maximal score; ties were resolved by minimal GO DAG distance to the corresponding anchor set (with additional distance-profile tie-breaks to avoid fixed category precedence). Genes with no GO annotations were labeled as “Unknown,” and genes with GO annotations not mapping to any anchor set were labeled as “Other”. TF node colors were assigned using a two-layer strategy. First, TFs were classified by their own GO annotations using the same score-and-distance rule; this assignment was accepted only when the top class was unambiguous. If the TF classification was ambiguous, “Other,” or “Unknown,” TF color was inferred from its targets using an evidence-weighted program score per class defined as the sum of target gene class scores (TFScore(C) = Σ score_g(C)). TFs were assigned to the class with maximal TFScore; ties were resolved using aggregated target-to-anchor distances and, only if necessary, a deterministic final tie-break.

### 2.8 Analysis of pro-inflammatory cytokines in supernatant

The CBA assay (BD Biosciences, #551811) was performed according to the manufacturer’s instructions. Briefly, 2 µL of each capture bead (IL-8, IL-1β, IL-10, IL-6, TNF-α and IL-12 p70) was added into a 1.5 mL microcentrifuge tube. Subsequently, 11 μL of each test supernatant was mixed with 11 μL of the prepared bead mixture and 11 μL of PE Detection Reagent. Samples were incubated for 3 hours at room temperature in darkness. After incubation, 200 μL of Wash Buffer was added to each sample and centrifuged at 1,600 rpm for 5 minutes at room temperature. The supernatant was carefully aspirated and discarded, leaving 50 uL of residual volume in each tube. Beads were gently resuspended, and an additional 200 μL of Wash Buffer was added. Samples were acquired immediately on a BD FACSVerse flow cytometer (BD Biosciences), and data were analyzed using FCAP Array software v3.0.1 (BD Biosciences). Human IL-11 concentrations in cell culture supernatants were measured using a commercial ELISA kit (Quantikine^®^, R&D Systems, Cat. No. D1100), following the manufacturer’s protocol. Cell culture supernatants were centrifuged at 1,000 x g for 15 minutes at 4°C to remove debris, and 50 μL samples were diluted 1:10 in calibrator diluent before analysis. Next, 100 µL of assay diluent and 100 µL of standards or samples were added to pre-coated wells and incubated for 2 h at room temperature. After 4 washes, 200 µL of Human IL-11 horseradish peroxidase conjugated-antibody were added to each well and incubated for 2.5 h at room temperature. The wells were washed and incubated with 200 µL of substrate solution for 30 min in the dark, and the reaction was stopped with 50 µL of stop solution. Finally, the optical density of each well was read at 450 nm with correction at 540 nm using the Synergy HTX microplate reader. IL-11 concentration was calculated from the standard curve using a lineal regression model.

### 2.9 Western blot

Cells were lysed at indicated time points using lysis buffer containing 100 mM NaCl, 10 mM Tris HCl pH 7.5, 0.5% NP-40, 1 mM EDTA and 1X protease inhibitors cocktail (Cat A32955, Pierce Thermo Scientific). Protein concentration was determined using the Protein Assay Dye Reagent Concentrate (Cat 5000006, Bio-Rad) following manufacturer’s instructions. 30 μg proteins were denatured in 1X Laemmli buffer at 95°C for 10 minutes and loaded onto 5-15% acrylamide gels for 2 hours at 120V. Proteins were transferred to a nitrocellulose membrane for 2 hours at 0.4A, 4°C. Membranes were blocked in 5% blocking solution (Cat 1706404, Bio-Rad) for at least 1 hour at room temperature followed by 3 washes with TBS-Tween. Primary antibodies were incubated at the appropriate dilution overnight at 4°C, washed 3 times with TBS-Tween and incubated with HRP-conjugated secondary antibodies (1:5000, Jackson ImmunoResearch) in 0.5% blocking solution for 2 hours at room temperature. Membranes were finally washed prior to chemiluminescent detection using the Western Blotting substrate (Cat 32106, Pierce Thermo Scientific) or the Immobilon Forte Western HRP substrate (Cat WBLUF0500, Millipore). Chemiluminescent images were acquired with the Alliance Q9 Advanced imaging system (Uvitec). Primary antibodies from Proteintech used in this study were: RIG-I (1:1000, Cat 20566-1-AP) and MDA-5 (1:3000, Cat 21775-1-AP). Antibody against cleaved IL-1β was purchased from Cell Signaling Technology (1:1000, Cat #83186). The following primary antibodies were purchased from Santa Cruz Biotechnology: Iba-1 (1:200, sc-398406), IRF-3 (1:500, sc-33641), RELA/NF-κB p65 (1:100, sc-515045), NF-κB p50 (1:200, sc-8414), Lamin A (1:1000, sc-71481), β-tubulin (1:1000, sc-365791), MAVS (sc-166583) and β-actin (1:3000, sc-47778). The following reagents were obtained through BEI Resources, NIAID, NIH: HIV-1 Nef antiserum (1:1000, ARP-2949), HIV-1 Vpu antiserum (1:1000, ARP-969), HIV-1 Vif antiserum (1:1000, ARP-809) and Monoclonal Anti-HIV-1 p24 (1:2000, Cat ARP-3537).

### 2.10 Indirect immunofluorescence

For IF experiments, 50,000 C20 cells were seeded in 24-well plates with 12 mm coverslips. Infections and treatments were performed as indicated. Cells were fixed at room temperature in 4% methanol-free PFA (Cat 28908, Pierce Thermo Scientific) for 20 minutes, washed three times with 1x PBS, treated with 0.1 M Glycine solution for 10 minutes, washed and permeabilized with 0.2% Triton X-100 for 5 minutes. Coverslips were then incubated in 1X Blocking solution (DIG Wash and Block Buffer Set Cat 11585762001, Roche) for 30 minutes. Primary and Alexa-Fluor conjugated secondary antibodies diluted in 1X Blocking solution were incubated for 1 hour each at 37°C in a humid chamber. Primary and Alexa-Fluor conjugated secondary antibodies were incubated for 1 hour each at 37°C. Cells were washed twice with 1X PBS, incubated with 1X DAPI (Cat D1306, Invitrogen) for 5 minutes at room temperature, washed three times with 1X PBS, three times with water, air-dried and mounted with aqueous Fluoromont mounting medium (Cat F4680, Sigma-Aldrich). Primary antibodies used for IF were: Iba-1 (1:50, sc-398406) and RELA/NF-κB p65 (1:50, sc-515045). Monoclonal Anti-HIV-1 p24 (1:200, ARP-3537) and Polyclonal Anti-HIV-1 p17 Protein (1:200, ARP-4811) were obtained from BEI Resources, NIAID. Secondary Alexa-Fluor conjugated antibodies were purchased from Invitrogen, ThermoFisher Scientific and used at 1:500 dilution. Images were obtained with an inverted OLYMPUS IX73 epifluorescence microscope (40X objective) or with a Nikon C2+ Confocal microscope (60X objective).

### 2.11 Statistical analyses

The statistical data analysis and graphics (mean ± standard deviation) were conducted using the GraphPad v10 program (La Jolla, CA 92037, USA). Unpaired two-tailed Student *t*-test, one-way ANOVA or two-way ANOVA (Dunnet’s or Bonferroni post-test) were performed considering p<0.05 as statistically significant.

## 3 Results

### 3.1 Transcriptional reprogramming during active HIV-1 replication in human microglia differs from a classical antiviral response

**Figure 1:**
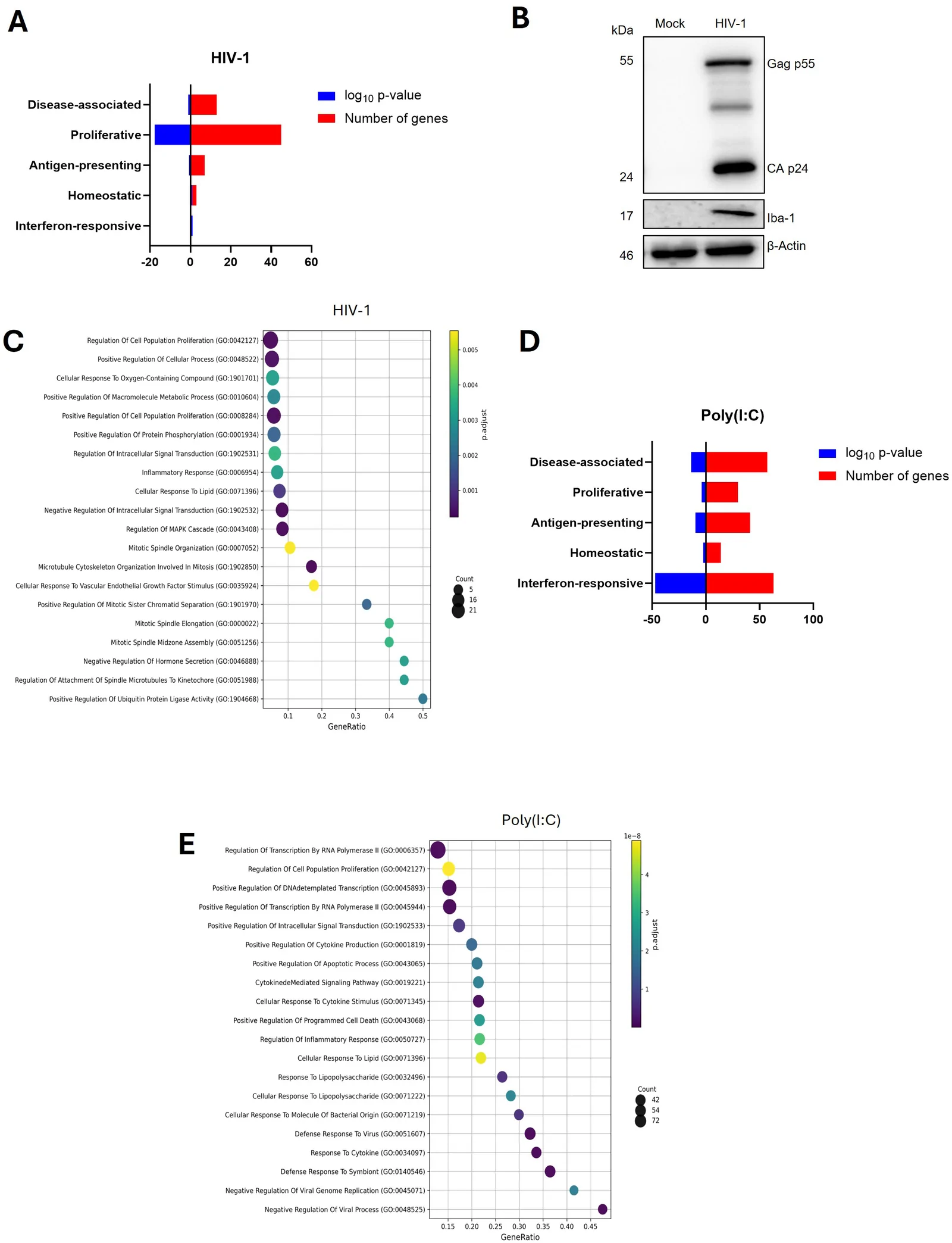
HIV-1 infection of human microglia does not induce a type-I interferon response. **(A)** Analysis of up-regulated genes associated with different microglia states in cells infected with HIV-1 for 2 days. Bars represent the number of genes (red) and the hypergeometric p-value (blue). **(B)** Expression of structural viral protein Gag and Iba-1 in uninfected (Mock) and HIV-1 infected microglia was evaluated by western blot. β-actin served as a loading control. **(C)** Gene ontology (GO) analysis of up-regulated genes from RNA-sequencing experiments showing the top twenty GO identified in cells infected with HIV for 2 days (left). **(D)** Analysis of up-regulated genes associated with different microglia states in cells transfected with poly(I:C) for 4 hours. Bars represent the number of genes (red) and the hypergeometric p-value (blue). **(E)** Gene ontology (GO) analysis of up-regulated genes from RNA-sequencing experiments showing the top twenty GO identified in cells transfected with poly(I:C).

Microglia are active cells able to react and adopt transcriptional signatures defining functional states including homeostatic, disease-associated, antigen-presenting, interferon-responsive or proliferative (35, 36). Since the adoption of these states is dependent on the environment to which microglia are exposed, we performed bulk RNA sequencing to characterize the reactivity of a human adult microglia cell line (C20 cells) to acute HIV-1 replication. Differential gene expression analysis of the RNA sequencing data identified 394 upregulated and 389 downregulated protein-coding transcripts in response to acute HIV-1 replication (**Supplementary Figure S1A**; **Supplementary Table 2**). Further analyses revealed that HIV-1 infection upregulates transcripts mostly associated with a proliferative state (e.g., cyclin B1, PLK1 and UBE2S) followed by transcripts defining a disease-associated state (e.g., CXCL8 and NCEH1) (**Figure 1A**; **Supplementary Table 3**). We also observed a potent induction of the myeloid marker ionized calcium-binding adaptor molecule 1 (Iba-1) in infected microglia (**Figure 1B**; **Supplementary Figure S1B**), suggesting reactivity to HIV-1 infection (37). In agreement with this notion, Gene Ontology analyses suggest that cell proliferation and mitosis are amongst the top ten processes upregulated by HIV-1 infection (**Figure 1C**). Since transcripts defining antigen-presentation or interferon-responsive states, which should be expected during a viral infection, were not widely represented in HIV-1-infected microglia, we analyzed transcriptional reprogramming of human microglia in response to the viral RNA analog polyinosinic:polycytidylic acid [poly(I:C)], observing the upregulation of 1,677 and the downregulation of 418 protein coding transcripts (**Supplementary Figure S1C**; **Supplementary Table 4**). In contrast to HIV-1 infection, poly(I:C)-transfected microglia presented upregulated transcripts characteristic of the interferon-responsive, disease-associated, and antigen-presenting states (**Figure 1D**; **Supplementary Table 5**). Consistent with this, Gene Ontology analyses revealed that poly(I:C) transfection mostly resulted in the induction of biological processes related to an antiviral response (**Figure 1E**; **Supplementary Table 6**). In agreement with these observations, we could not observe the induction of the interferon beta (IFN-β) mRNA in C20 cells infected for 48 hours with VSVg-pseudotyped HIV-1 isolates NL4.3, LAI and ADA or with the HIV-2 isolate Rod10 while such an induction was readily observed in microglia infected with Zika virus (**Supplementary Figure S1D–F**).

Together, these results show that acute HIV-1 replication in a human adult microglia cell line results in a transcriptional reprogramming that differs from a classical antiviral response triggered by the sensing of non-self RNA.

### 3.2 HIV-1 activates the NF-κB signaling pathway and inflammatory mediators during infection

**Figure 2:**
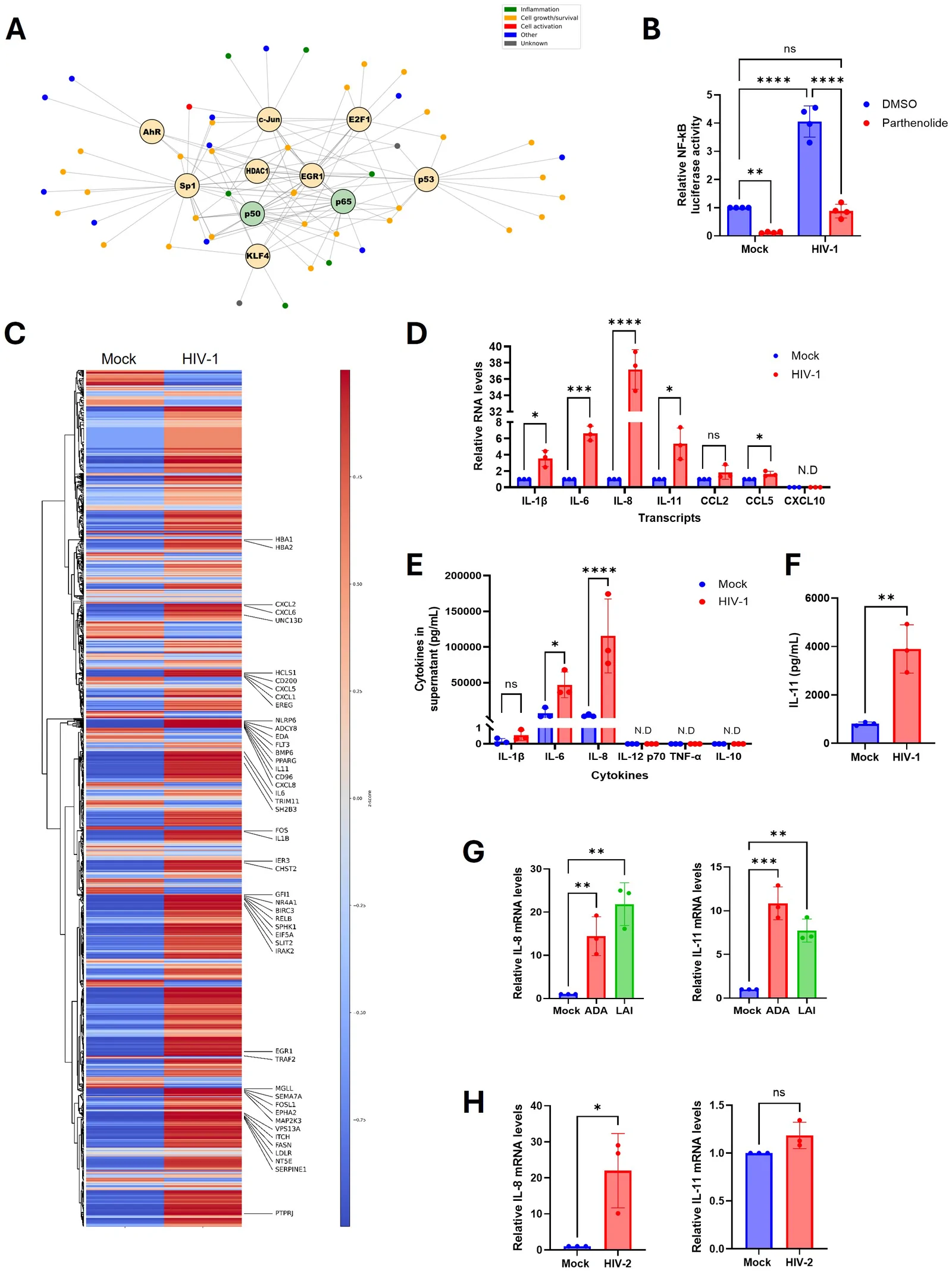
HIV-1 infection activates the NF-κB signaling pathway and upregulates inflammatory cytokines IL-8 and IL-11 in human microglia. **(A)** Transcriptional regulatory network analysis showing the top 10 transcription factors (TFs) associated with HIV-induced up-regulated genes. Nodes (TFs) and circles (genes) are colored by cellular processes: Green (Inflammation), Yellow (Cell growth/survival), Red (Cell activation), Blue (Other) and Grey (Unknown). **(B)** Reporter cell line carrying five copies of the canonical NF-κB binding sites controlling the expression of the luciferase reporter gene were mock-treated or infected with HIV-1 for 24 hours and then treated with 10 μM NF-κB inhibitor parthenolide for additional 24 hours. DMSO was used as the vehicle control. Firefly luciferase activity was measured and normalized relative to mock cells treated with DMSO. Data represent mean ± SD of four biological replicates. **(C)** Hierarchical clustering of genes associated to inflammation annotated within the “Inflammatory response” (GO:0006954) and “Cytokine-mediated signaling pathway” (GO:0019221) obtained from RNA-sequencing data of mock-treated and HIV-1 infected microglia for 2 days. Statistically significant up-regulated genes induced by HIV-1 are indicated in the heatmap. **(D)** Validation of HIV-1 induced up-regulation of pro-inflammatory cytokines IL-1β, IL-6, IL-8, IL-11, CCL2, CCL5 and CXCL10 at the mRNA level by RT-qPCR. N.D. non-detected. Data represent mean ± SD of three biological replicates. **(E, F)** Levels of secreted pro-inflammatory cytokines IL-1β, IL-6, IL-8, IL-12 p70, TNF-α, IL-10 **(E)** and IL-11 **(F)** in the supernatant of mock and HIV-1 infected microglia measured by CBA and ELISA, respectively. Data represent mean ± SD of three biological replicates. **(G, H)** RT-qPCR analysis of IL-8 (left) and IL-11 (right) relative mRNA levels in cells infected with different HIV-1 strains (ADA, LAI) **(G)** or with HIV-2 **(H)**. Data represent mean ± SD of three biological replicates (ns, not significant; *p < 0.05; **p < 0.01; ***p < 0.001; ****p < 0.0001).

We then wanted to better characterize the cellular response of human microglia towards HIV-1 replication. A look at the upregulated transcripts data set allowed us to identify a transcriptional regulatory network (TRN) in which KLF4, RelA (p65), p53, NFKB1 (p50), E2F1, Sp1, HDAC1, AhR, JUN (c-Jun) and EGR1 were the top ten transcriptional regulators driving the response of microglia to HIV-1 infection (**Figure 2A**; **Supplementary Table 7**). From these, we were mostly interested in p65 and p50, the two subunits of the canonical NF-κB transcription factor, as they were classified as inflammatory transcription factors within the identified TRN while the remaining were mostly associated to cell proliferation and survival (**Figure 2A**). However, analyses of p65 localization by both cellular fractionation and immunofluorescence staining revealed a marginal nuclear translocation of the NF-κB subunit in microglia infected either for 24 or 48 hours as compared in cells transfected with poly(I:C) (**Supplementary Figure S2A**). We also failed to detect p65 nuclear translocation at 1, 3, 6 and 12 hours post-infection (**Supplementary Figure S2B**). Therefore, to have a more sensitive readout of the NF-κB signaling activation, we generated a microglia reporter cell line in which five canonical κB-binding sites command the transcription of the firefly luciferase reporter gene. Consistent with the TRN identified, we observed that HIV-1 infection resulted in a significant increase of the luciferase activity of the reporter microglia that was blocked by the p65 translocation inhibitor parthenolide (**Figure 2B**), indicating activation of the canonical NF-κB signaling pathway during infection. Of note, the effects of parthenolide on the HIV-1-induced activation of the NF-κB reporter were not due to any impact on viral replication as judged by unchanged levels of viral transcripts (**Supplementary Figure S2C**). For comparison, we also generated a reporter microglia cell line in which RelB command the expression of the luciferase reporter gene to evaluate the activation of the non-canonical NF-κB signaling pathway, observing that the basal activity of the RelB reporter was inhibited by HIV-1 infection (**Supplementary Figure S2D**).

Given the well-described roles of the canonical NF-κB transcription factor as a master regulator of the pro-inflammatory response (38–40), we sought to characterize the pro-inflammatory response triggered by HIV-1 infection in this microglia model. An inspection of the gene ontology terms issued from our dataset of transcripts up-regulated by HIV-1 identified “Inflammatory response” (GO:0006954) and “Cytokine-mediated signaling pathway” (GO:0019221) amongst the top 25 terms characterizing the reactivity of human microglia to infection (**Supplementary Table 6**). We used these GO terms and curated a panel of 1,513 inflammation-related genes, of which 989 were detectably expressed in our dataset. From this panel, we identified 48 transcripts that were significantly upregulated upon HIV-1 infection (**Figure 2C**; **Supplementary Table 8**). On the other hand, transfection of poly(I:C) results in a transcriptional reprogramming through a TRN mostly regulated by transcription factors RelA (p65), NFKB1 (p50), c-Jun, STAT3, STAT1, p53, HDAC1, BRCA1, SIRT1 and ATF4, which results in the induction of 218 inflammation-related genes (**Supplementary Figures S2E, F**; **Supplementary Tables 9**, **10**). To verify the induction of pro-inflammatory genes by HIV-1 infection, we conducted RT-qPCR analyses and confirmed a significant increase in the mRNA levels of IL-1β, IL-6, CXCL8 (IL-8) and IL-11 while CCL5 was marginally induced, CCL2 experimented no changes and CXCL10 was not detected (**Figure 2D**). In contrast, poly(I:C) transfection induced all the transcripts analyzed with a potent induction of CCL5 and CXCL10 (**Supplementary Figure S2G**). Quantification of a panel of pro-inflammatory cytokines in the supernatants of uninfected and HIV-1-infected microglia by Cytometric Bead Array (CBA) revealed a significant increase in the secretion of IL-6 and IL-8 but not IL-1β, IL-12 p70, TNF-α or IL-10 (**Figure 2E**). Since we observed an increase in the relative levels of the IL-1β mRNA without the expected increase in the secreted protein, we looked at the intracellular IL-1β protein by western blot observing the presence of pro-IL-1β but not the processed form (**Supplementary Figure S2H**). We also analyzed the secretion of IL-11 in HIV-1-infected microglia as it was, together with IL-8, amongst the highest up-regulated pro-inflammatory transcripts in our data sets (**Supplementary Figure S1B**; **Supplementary Table 2**). As such, quantification of IL-11 by ELISA confirmed an increased secretion of this cytokine to the supernatants of infected microglia (**Figure 2F**). Since IL-8 and, at a lesser extent, IL-6 have been associated with inflammation in PLWH including patients with neurocognitive impairments and IL-11 has emerged as an important player in inflammatory diseases including CNS pathologies (41–45), we looked at the conservation of their induction during infection of microglia by other human lentiviruses. As such, while the induction of IL-8 and IL-11 mRNA expression was also observed in microglia infected with the HIV-1 isolates ADA and LAI, infection with the related virus HIV-2 only resulted in the increase of IL-8 but not IL-11 (**Figures 2G, H**), suggesting that these cytokines might be induced by different mechanisms during infection.

Together, these results indicate that HIV-1 replication in human microglia triggers the activation of the canonical NF-κB signaling pathway and induces a subset of cytokines with reported neuroinflammatory properties, such as IL-8 and IL-11.

### 3.3 Different mechanisms drive the induction of IL-8 and IL-11

**Figure 3:**
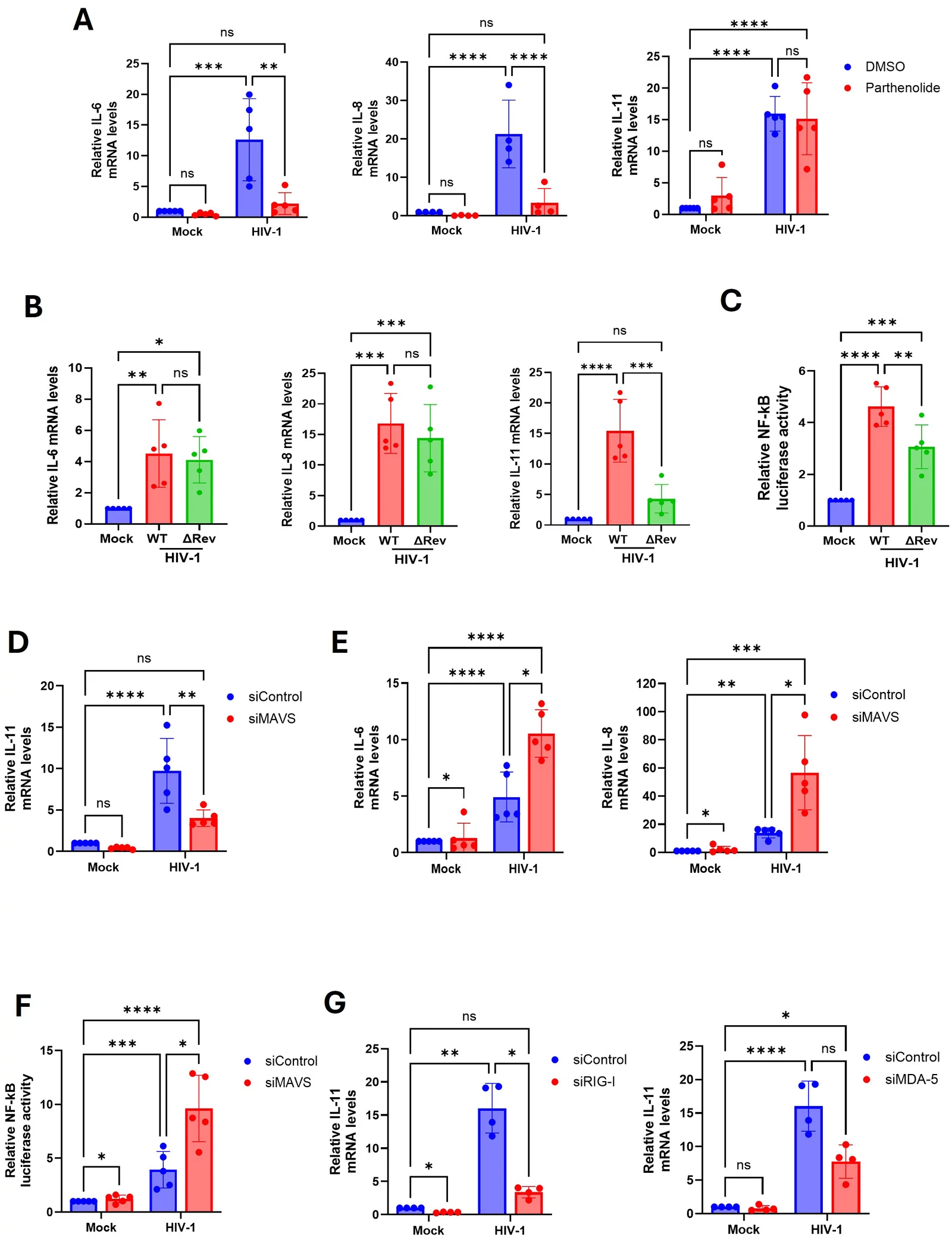
HIV-1-induced IL-11, but not IL-8 expression is dependent on sensing of intron containing viral RNAs and signaling of RIG-I-like receptors (RLRs). **(A)** Cells were mock-treated or infected with HIV-1 for 24 hours and then treated with 10 μM NF-κB inhibitor parthenolide for additional 24 hours. DMSO was used as the vehicle control. RT-qPCR analysis of IL-6 (left), IL-8 (middle) and IL-11 (right) mRNA levels relative to mock cells treated with DMSO. Data represent mean ± SD of five biological replicates. **(B, C)** Analysis of relative IL-6 [**(B)** left], IL-8 [**(B)** middle], IL-11 [**(B)** right] mRNA levels and NF-κB luciferase reporter activity **(C)** in cells infected with equal RNA copy numbers of Wild Type (WT) HIV-1 or a replication-deficient mutant virus lacking Rev expression (ΔRev). Data represent mean ± SD of five biological replicates. **(D–F)** Cells were transfected with non-targeting control siRNA (siControl) or siRNA targeting MAVS (siMAVS) for 24 hours prior infection with HIV-1 for 2 days. Induction of IL-11 **(D)**, and IL-8 **(E)** relative mRNAs levels were evaluated by RT-qPCR. **(F)** Cells transfected with an siRNA targeting MAVS were infected for 2 days. Activation of the NF-κB signaling pathway was analyzed by measuring the firefly luciferase activity normalized to non-infected mock cells transfected with siRNA control. Data represent mean ± SD of five biological replicates. **(G)** Cells were transfected with siRNAs against the RLRs RIG-I (siRIG-I, left) or MDA-5 (siMDA-5, right) for 24 hours prior infection with HIV-1 for 2 days. Analysis of IL-11 mRNA expression was performed by RT-qPCR. Data represent mean ± SD of four biological replicates. Statistical significance is indicated as: ns, not significant; *p < 0.05; **p < 0.01; ***p < 0.001; ****p < 0.0001.

We then sought to identify the mechanism by which HIV-1 infection was triggering the activation of the pro-inflammatory response. Interestingly, treatment of cells with the NF-κB signaling inhibitor parthenolide abolished the induction of IL-6 and IL-8 but not IL-11 (**Figure 3A**), further indicating that these pro-inflammatory cytokines are induced by different mechanisms. We then focused on the sensing of the Rev-dependent intron-containing RNA (i.e., unspliced (US) and partially spliced (PS) transcripts) shown as the trigger of immune activation during HIV-1 infection in monocyte-derived dendritic cells (MDDC), monocyte-derived macrophages (MDM), and monocyte-derived-microglia (MMG) (16, 26–29). For this, we took advantage of a mutant virus not expressing the Rev protein (ΔRev), which is only able to express completely spliced (CS) transcripts and derived proteins such as Nef (**Supplementary Figure S3A**). Interestingly, while the ΔRev virus was still able to induce the transcripts encoding IL-6 and IL-8, it failed to induce the IL-11 mRNA (**Figure 3B**), suggesting that sensing of intron-containing RNA and downstream signaling pathways are a major contributor for the induction of IL-11 but not IL-6 and IL-8. Consistent with the ability to induce the IL-6 and IL-8 mRNA, the ΔRev virus was also able to induce the NF-κB reporter microglia (**Figure 3C**), suggesting that activation of the NF-κB signaling pathway does not involve the sensing of Rev-dependent transcripts nor a viral protein encoded by them.

Since Rev-dependent transcripts in MDM and MDDC are sensed by melanoma differentiation-associated protein 5 (MDA5) leading to the activation of MAVS signaling (28, 29), we evaluated the involvement of this RNA-sensing pathway in the induction of IL-11. Consistent with a mechanism involving the sensing of intron-containing RNA, silencing of MAVS resulted in a significant reduction in the induction of the IL-11 mRNA triggered by HIV-1 infection (**Figure 3D**). However, MAVS silencing resulted in a slight but significant increase in the induction of the IL-6 and IL-8 transcript and the NF-κB reporter (**Figures 3E, F**), indicating that MAVS signaling is not involved in the activation of the NF-κB signaling pathway by HIV-1. Of note, we did not observe a major effect of MAVS silencing on viral RNA or proteins (**Supplementary Figure S3B**), indicating that the defects on IL-11 induction were not due to decreased levels of intron-containing transcripts. We also analyzed any involvement of Toll-like receptors (TLRs) by silencing MyD88, the adapter for the signaling of all TLRs (46, 47), but we could not observe any effect on the NF-κB reporter nor in IL-11 transcript levels (**Supplementary Figures S3C, D**). Since MyD88 was recently shown to contribute to TLR3-dependent NF-κB activation in macrophages (47), we also tested the small molecule CU-CPT 4a, which inhibits dsRNA binding by TLR3. Once again, we could not observe any impact of CU-CPT 4a on the HIV-1-mediated induction of the NF-κB reporter nor the levels of the IL-11 transcript (**Supplementary Figures S3E, F**). We then silenced RLRs, RIG-I and MDA5, to identify the RNA sensor of the HIV-1 US/PS RNA in this microglia model. In agreement with an induction independent of viral RNA sensing, knockdown of RIG-I or MDA5 has no major effects on the induction of the IL-8 mRNA nor the activation of the NF-κB signaling pathway in HIV-1-infected microglia (**Supplementary Figures S3G, H**). However, we observed that induction of the IL-11 mRNA by HIV-1 was largely dependent on RIG-I and, to a lesser extent, MDA5 (**Figure 3G**), suggesting that RIG-I might be the main RNA sensor of the HIV-1 intron-containing RNA in our cellular model. No effects on viral RNA and proteins were observed upon RIG-I or MDA5 knockdown (**Supplementary Figure S3I**), suggesting that defects on IL-11 induction were not due to decreased levels of intron-containing RNA.

Together, these data indicate that induction of IL-6/8 and IL-11 are regulated by different mechanisms, with IL-6 and IL-8 being induced by activation of the NF-κB signaling pathway and IL-11 being mainly induced by sensing of intron-containing RNA and activation of RIG-I/MAVS signaling.

### 3.4 Activation of the NF-κB signaling and induction of IL-6 and IL-8 depends on the viral protein Nef

**Figure 4:**
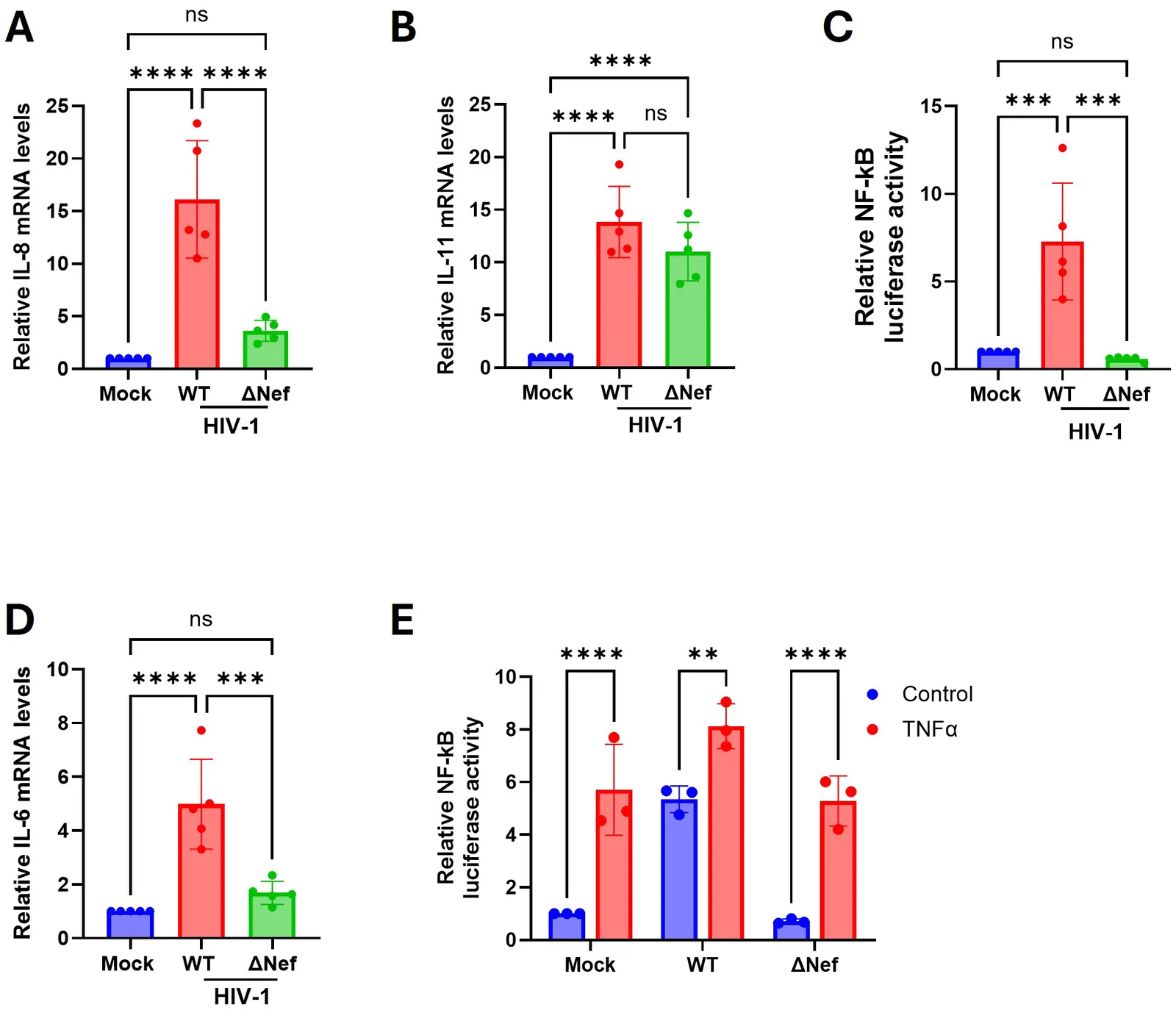
HIV-1 Nef drives NF-κB activation and IL-8 induction in human microglia. **(A–D)** Analysis of relative IL-8 **(A)**, IL-11 **(B)** mRNA levels, NF-κB luciferase reporter activity **(C)** and IL-6 **(D)** mRNA levels in cells infected with equal RNA copy numbers of Wild-Type (WT) HIV-1 or a Nef-deficient mutant (ΔNef). Data are presented as mean ± SD of five biological replicates. **(E)** Cells were infected with HIV-1 WT or ΔNef for 2 days and stimulated with 10 ng/mL TNF-α for 6 hours prior NF-κB reporter activity determination. Treatment with ultra-pure water was used as vehicle control. Data are presented as mean ± SD of three biological replicates. Statistical significance is indicated as: ns, not significant; **p < 0.01; ***p < 0.001; ****p < 0.0001.

Then, we sought to determine the mechanism underlying NF-κB signaling pathway activation and IL-8 induction. Data obtained with the ΔRev virus suggest that a viral product encoded by a completely spliced transcript is sufficient to activate this arm of the pro-inflammatory response triggered by HIV-1 infection in microglia. We focused on the accessory protein Nef as it has previously been shown as an activator of the canonical NF-κB signaling pathway (48–51). Interestingly, a virus lacking Nef failed to induce IL-8 transcript levels while preserving its ability to induce IL-11 (**Figures 4A, B**). Of note, the ΔNef virus expresses viral transcripts and proteins at similar levels to the wild type virus (**Supplementary Figures S4A, B**). Moreover, the ΔNef virus was unable to activate the NF-κB reporter in microglia (**Figure 4C**), indicating that this accessory protein is responsible for the activation of the NF-κB signaling pathway and the induction of IL-8 in these cells. Induction of IL-6 was also dependent on the presence of Nef and the activation of the NF-κB signaling pathway, indicating that these two pro-inflammatory cytokines are induced by the same mechanism (**Figure 4D**). Interestingly, we observed that the ΔNef virus does not interfere with the TNF-α-induced activation of the NF-κB signaling pathway (**Figure 4E**), suggesting that other viral proteins do not counteract the activation of this signaling pathway by Nef.

Together, these data indicate that HIV-1 activates the NF-κB signaling pathway through the accessory protein Nef leading to the induction of the pro-inflammatory cytokine IL-8.

### 3.5 Pro-inflammatory cytokines correlate with viral loads in the cerebrospinal fluid of PLWH

**Figure 5:**
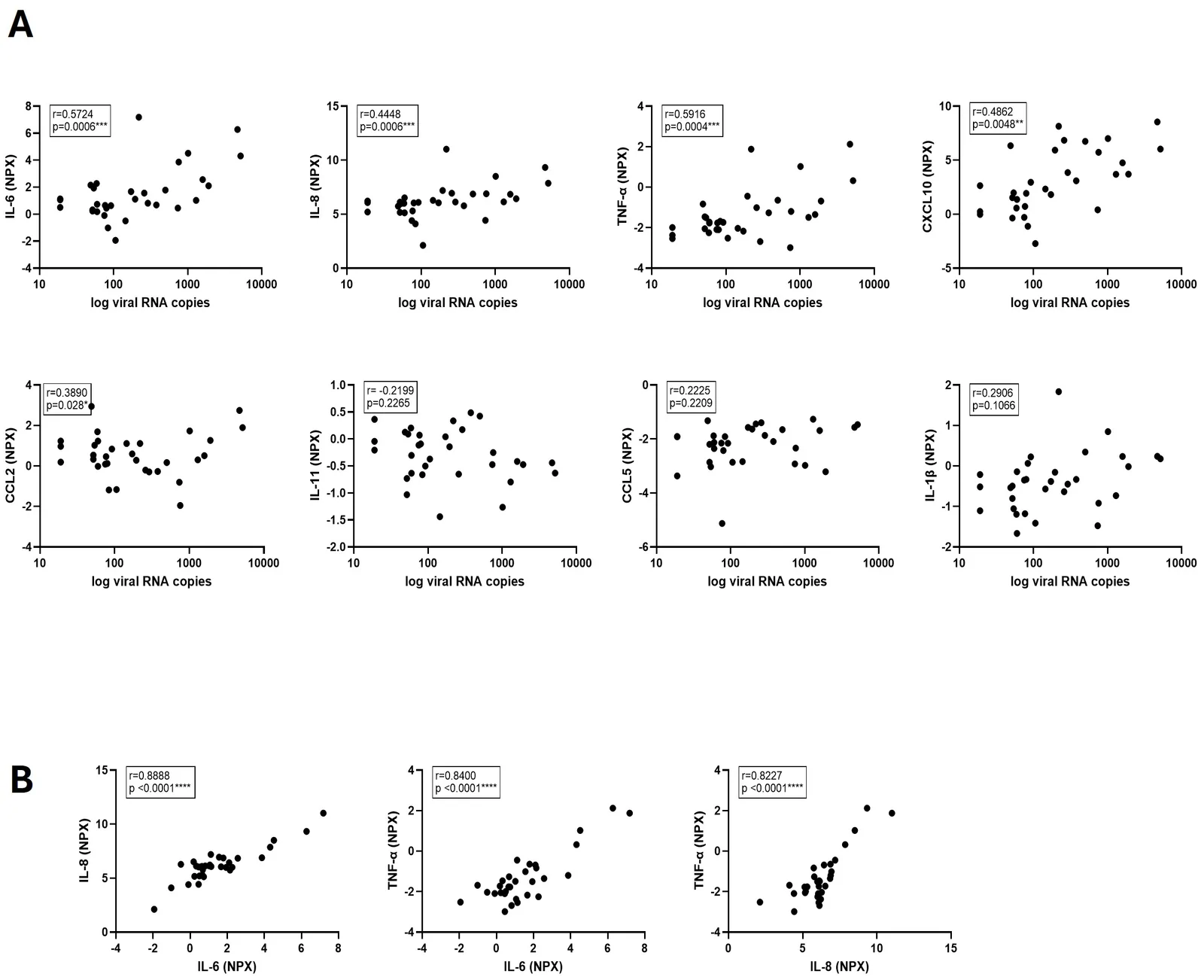
Levels of proinflammatory cytokines in cerebrospinal fluid correlate with active HIV-1 replication in the central nervous system despite suppressive ART. **(A)** Correlation analyses of IL-6, IL-8, TNF-α, CXL10, CCL2, IL-11, CCL5, IL-1β levels and viral RNA copies in cerebrospinal fluid of people living with HIV-1 with undetectable viral load in plasma serum. **(B)** Correlation between levels of IL-6 and IL-8, IL-6 and TNF-α as well as IL-8 and TNF-α. Cytokine levels are reported in NPX units as described in (52).

Finally, we sought to evaluate whether our findings during acute HIV-1 replication in a microglia cell line correlate with viral replication and inflammation in the CNS of PLWH. For this, we leverage publicly available data obtained from a multicenter cohort of PLWH, which includes proteomic and viral loads information from the CSF of the participants (52). Therefore, we selected patients having viral loads in the CSF higher than the threshold of 19 copies/mL established by the authors (regardless the patients were under cART) and used their proteomic data to evaluate the correlation between the levels of viral replication (i.e., viral loads) and pro-inflammatory cytokines. Interestingly, we observed that the levels of IL-6, IL-8, TNF-α, CXCL10 and CCL2, but not IL-11, CCL5 and IL-1β presented significant correlations with viral loads (**Figure 5A**), suggesting that active replication in microglia contributes to acute inflammation represented by an increase in the levels of IL-6, IL-8, TNF-α, CCL2 and CXCL10 in CSF while other cytokines might reflect persistent low-grade inflammation in the CNS. We also observed potent correlations between IL-6 with IL-8 and TNF-α as well as IL-8 with TNF-α (**Figure 5B**), probably suggesting that these cytokines are induced together due to active viral replication.

Together, these analyses suggest that a subset of pro-inflammatory cytokines including IL-6 and IL-8 are induced by active HIV-1 replication in the CNS while others pro-inflammatory markers may represent a state of chronic neuroinflammation occurring independently of viral replication.

## 4 Discussion

Persistent immune activation and chronic inflammation have been widely reported in PLWH (53–55). Activation of myeloid cells including dendritic cells, monocytes and macrophages during chronic HIV-1 infection has been reported as a key contributor to systemic inflammation (56, 57). For instance, HIV-1 infection activates monocytes and macrophages leading to the secretion of pro-inflammatory mediators such as sCD14 and sCD163 as well as TNF-α, IL-1β and IL-6 (58–62), which are linked to the development of cardiovascular disease and other comorbidities in PLWH even under suppressive antiretroviral therapy (ART) (63–68). HIV-1 also resides in the brain causing inflammation, neuronal damage and neurocognitive impairments (69–71). As such, markers in the cerebrospinal fluid including sCD14, sCD163, IFN-γ, IL-1α, IL-7, IL-8, sTNFR-II have been consistently associated with neurocognitive impairment in PLWH (41). Microglia, the sentinels of the central nervous system, are the main targets for HIV-1 infection in the brain being considered key contributors to inflammation and neurocognitive impairment in PLWH (5–9). Data presented here using a model of adult human microglia elucidate two distinct mechanisms leading to the induction of pro-inflammatory mediators during acute HIV-1 replication. As such, we observed that HIV-1-infected microglia adopted a transcriptional program defining a proliferative state together with the induction of a small subset of pro-inflammatory transcripts including IL-6, IL-8 and IL-11 amongst others. Consistent with the post-transcriptional sensing of intron-containing transcripts reported in monocyte-derived dendritic cells, macrophages and microglia (16, 26–29), we observed that activation of MAVS signaling by RIG-I-like receptors but not TLR7/8 nor TLR3 resulted in the induction of IL-11, a member of the IL-6 family of cytokines. Such induction of IL-11 was independent from the activation of NF-κB signaling, probably reflecting the activation of the MAVS/IRF5 signaling as was recently reported in MDMs (28). However, whether activation of the MAVS signaling involves IRF5 or the activation of other signaling pathways leading to the induction of IL-11 and other pro-inflammatory mediators requires further investigation. In this regard, viral proteins including Tat and gp120 have been reported to induce neuroinflammation in different cellular models (72). Importantly, IL-11 activates the STAT3 signaling pathway and has emerged as an important contributor in different disease contexts including neuroinflammatory diseases such as multiple sclerosis (73–75). Beyond STAT3 signaling, IL-11 also activates the ERK/MAPK pathway (76). In line with a potential role for this pathway in the inflammatory response observed, genes associated with the MAPK cascade regulation were enriched in the RNAseq data set of HIV-1-infected microglia. Additionally, ERK/MAPK signaling can promote IL-1β transcription and inflammasome priming (77). As shown in our results, the accumulation of pro-IL-1β in the absence of detectable mature IL-1β may therefore reflect transcriptional priming without robust inflammasome activation (78). These findings suggest that IL-11-associated MAPK signaling may be contribute to the observed inflammatory response although additional studies are required to establish this mechanism. Nevertheless, we observed that IL-11 levels do not correlate with viral loads in CSF of PLWH and thus, whether induction of IL-11 in microglia plays a role in HIV-1-mediated neuronal injury remains to be investigated. In a second mechanism, we demonstrate that the viral protein Nef activates the NF-κB signaling pathway leading to an increased synthesis and secretion of IL-6 and IL-8. Interestingly, previous studies using either ectopic expression of Nef or treatment of cell cultures with the recombinant protein have shown the ability of Nef to activate the NF-κB signaling pathway and/or induce the expression of IL-6 and IL-8 in different cellular models including microglia and astrocytes (48–51, 79), further stressing the ability of Nef to trigger pro-inflammatory cytokine responses. Still, the limited subset of pro-inflammatory transcripts identified in our analyses of HIV-1-infected microglia could probably reflect the counteracting activity of Vpu, which can block the activation of NF-κB (50, 80, 81). Nevertheless, we also observed that IL-6 and IL-8 levels presented a significant correlation with viral loads in the CSF of PLWH indicating that HIV-1 viral replication and therefore, Nef expression might contribute to increase levels of these pro-inflammatory mediators. Although TNF-α levels correlate with viral loads, IL-6 and IL-8 in the CSF of PLWH, we could not detect an increase in the levels of this cytokine in the supernatants of C20 cells infected with HIV-1 for 48 hours. Of note, TNF-α was shown to be induced within hours in macrophages treated with LPS (82, 83), therefore, whether Nef also induces TNF-α through the activation of NF-κB earlier during infection requires further investigation. Nef is well known as a critical factor for HIV-1 replication, immune escape and pathogenesis (84–87). For instance, Nef promotes viral replication and immune escape by downregulating the host restriction factor, SERINC5 (88) as well as CD4 and MHC-I from the cell surface (89, 90). Moreover, viruses with mutations in the *nef* gene have been identified in long-term nonprogressors individuals and Nef was shown to be critical to maintain high viral loads and induce AIDS in rhesus macaques infected with simian immunodeficiency virus (SIV) (86, 91–93). In addition, Nef-deficient HIV-1 cannot replicate efficiently and deplete CD4+ T-cells in humanized mice (94). Finally, expression of Nef alone can induce an AIDS-like syndrome as well as cardiomyopathy in animal models (95–98).

Although we acknowledge that the use of an immortalized adult human microglia cell line may represent a limitation in our study, we consider C20 cells as a reliable model to carry out mechanistic studies involving viral replication and immune activation in the brain as previously shown for HIV-1 uncoating, integration and latency (15, 17, 99, 100). Moreover, a recent study comparing the pro-inflammatory profiles of primary and iPSC-derived microglia treated with LPS, IL-1β, TNF-α, IFN-γ revealed different basal pro-inflammatory states and responses. As such, human primary microglia presented low basal levels of inflammatory markers and induced the secretion of CXCL10 (IP-10), IL-8, IL-6, CCL2 (MCP-1) and CCL5 (RANTES) but no other pro-inflammatory mediators such as ICAM-1, VCAM-1, CX3CL1 (Fractalkine), G-CSF and GM-CSF in a treatment-specific manner (101). In contrast, iPSC-derived microglia were described as highly pro-inflammatory cells presenting high basal levels of ICAM-1, CX3CL1, IL-8, IL-6, G-CSF, GM-CSF and CCL2. As such, they only responded to LPS by inducing IL-6 and CCL5 and to IFN-γ by inducing CCL5 (101). Our RNA-seq and cytometric bead array (CBA) analyses in C20 cells showed a low basal pro-inflammatory state and the induction of a similar pattern of pro-inflammatory mediators including IL-6, IL-8, CCL2, CCL5, CXCL10 in a treatment (HIV-1 or poly(I:C))-dependent manner. Indeed, we showed that C20 cells presented different responses to HIV-1 infection and non-self RNA sensing stressing the usefulness of this cellular model to study specific virus – host interactions. Finally, it should be considered that adult primary microglia are very hard to obtain and must be maintained in a culture medium known to rapidly influence their transcriptional programs upon isolation (102, 103).

It is noteworthy that HIV-1 infection of C20 cells for 48 hours was not associated to the induction of a type-I interferon (IFN-I) response nor the induction of CXCL10, which is a classical marker for antiviral and inflammatory responses. Although these observations in our cellular model agree with several previous studies across different cell types including MDMs and dendritic cells (104–108), we could not discard a late IFN-I response as has been shown in macrophages (109). Indeed, we observed a correlation between CXCL10 levels and viral loads in the CSF of PLWH indicating that this cytokine is induced during active viral replication in the CNS. In this regard, it should be considered that recent studies have shown that HIV-1 uncoating occurs in the host cell nucleus, an event that may prevent the early sensing of the incoming RNA (110, 111). Moreover, accessory proteins including Nef, Vpu and Vpr have been shown to interfere with the establishment of a type-I interferon response in different cell types (105, 112–114), which may account for a delayed antiviral response in C20 cells. As such, we observed a potent induction of the CXCL10 transcript and several ISGs in cells transfected with polyI:C, confirming the ability of the C20 microglia model to mount a strong IFN-I antiviral response. Since treatment with recombinant human IFN-β results in a dramatic reduction of HIV-1 replication in this cellular model (data not shown) and infection in human brain organoids models have shown the induction of IFN-I responses (12–14), the interplay between HIV-1 and the microglia antiviral response microglia deserves further investigation.

Since HAND still occurs in approximately a half of the PLWH independently of suppressive antiretroviral therapy (115), the mechanisms proposed in this work can provide useful information for the development of pharmacological interventions aimed at reducing the pathogenesis caused by HIV-1 infection.

## Data Availability

RNA-seq data were uploaded at GEO (GSE318503). Plasmids and cell lines generated in this work will be available upon reasonable request.
