# Supplementary material for: NF-κB-dependent and independent inflammatory responses during acute HIV-1 infection in a model of human microglia
Source: Front Immunol. 2026 Aug 14;17:1912292. doi: 10.3389/fimmu.2026.1912292 (PMC13521827; doi:10.3389/fimmu.2026.1912292)
Supplement: Supplementary file 1 [file DataSheet1.pdf]

Supplementary Material

Supplementary Figures and Tables

For more information on Supplementary Material and for details on the different file types accepted, please see here.

1.1 Supplementary Figures

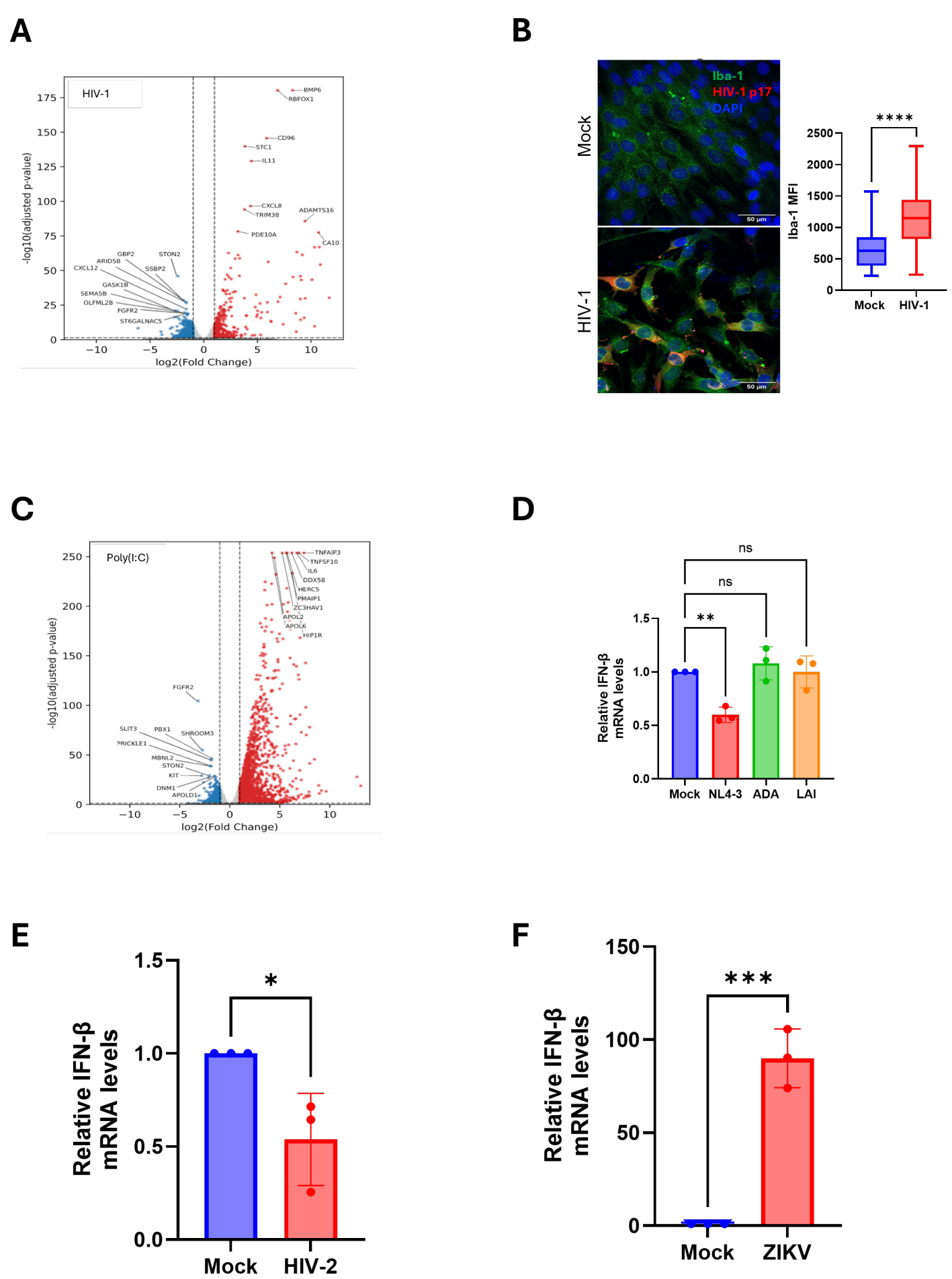

Supplementary Figure 1. Microglia activation in response to HIV infection occurs in the absence of a type-I interferon response.
(A) Volcano plot showing differentially expressed protein-coding genes induced by HIV-1 infection in microglia. Top ten up (red) and down-regulated (blue) genes are indicated. Data analysis was performed from n=2 independent experiments of RNA-sequencing. **(B)** Mean fluorescence intensity (MFI) quantification of Iba-1 signal in Mock and HIV-1 infected microglia from images shown in left panel **(B, right)**. Data represent mean ± SD of three biological replicates. (****, $p < 0.0001$). **(C)** Volcano plot showing differentially expressed protein-coding-genes in cells transfected with poly(I:C) versus control cells. Samples for RNA-sequencing were obtained from n = 3 independent experiments.

(D-F) C20 cells were infected with HIV-1 NL4-3, ADA, LAI **(D)**, HIV-2 **(E)** for 48 hours or Zika virus **(F)** for 24 hours and levels of the IFN-β mRNA were evaluated by RT-qPCR. Data was obtained from n=3 independent experiments and presented as mean ± standard deviation (ns, non-significative, *$p<0.05$, **$p<0.01$, ****, $p < 0.0001$).

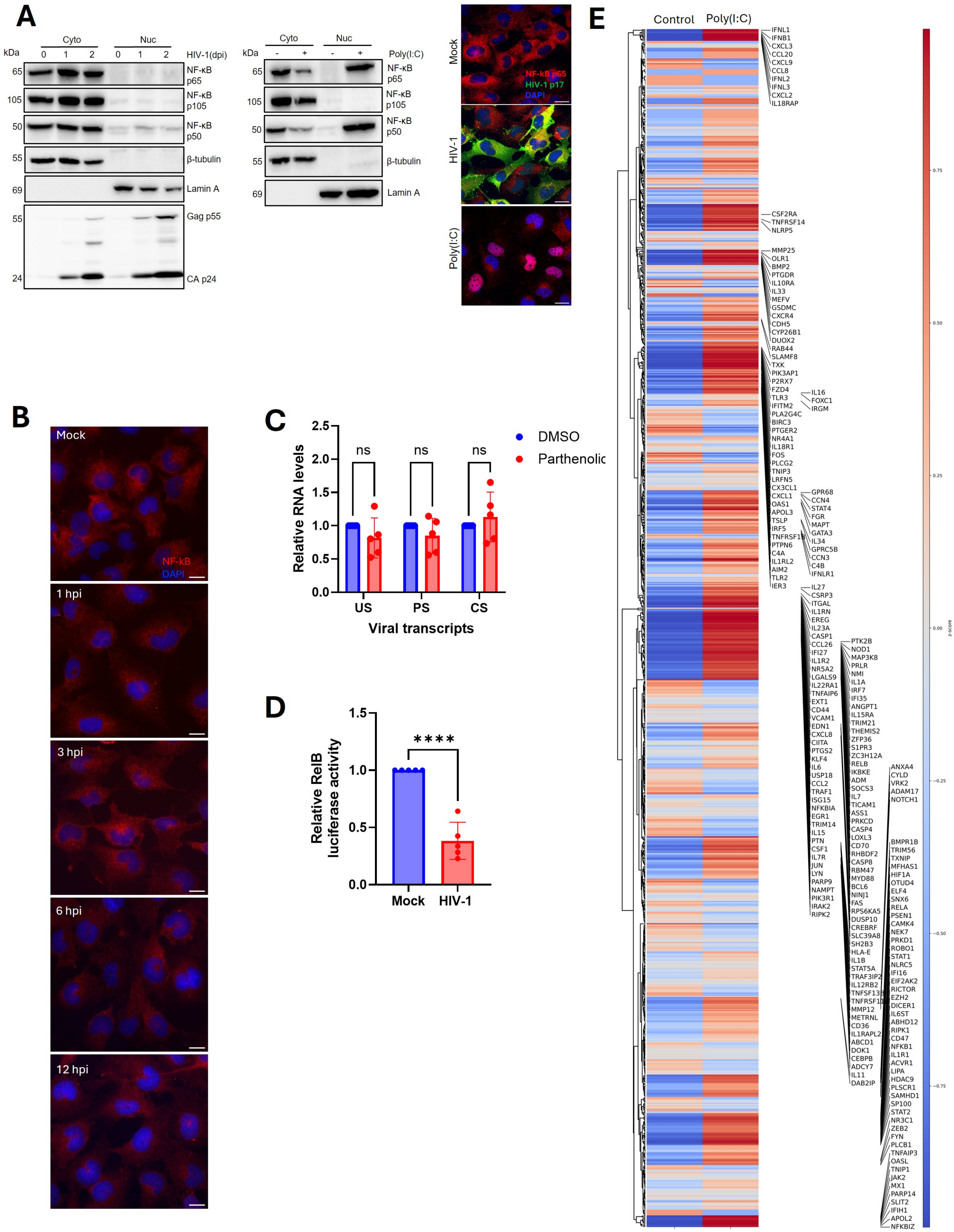
A
kDa
Cyto
Nuc
HIV-1(dpi)
0 1 2 0 1 2
65
NF-κB p65
105
NF-κB p105
50
NF-κB p50
55
β-tubulin
69
Lamin A
55
Gag p55
24
CA p24
Poly(I:C)
- + - +
Mock
HIV-1
NF-κB p65
HIV-1 p17
DAPI
B
Mock
1 hpi
3 hpi
6 hpi
12 hpi
NF-kB
DAPI
C
Relative RNA levels
2.5
2.0
1.5
1.0
0.5
0.0
ns
ns
ns
US
PS
CS
Viral transcripts
DMSO
Parthenolide
D
Relative RelB luciferase activity
1.5
1.0
0.5
0.0
****
Mock
HIV-1
E
Control
Poly(I:C)
IFNL1
IFNB1
CXCL3
CXCL9
CCL20
CCL8
IFNL2
IFNL3
CXCL2
IL18RAP
CSF2RA
TNFRSF14
NLRP5
MMP25
OLR1
BMP2
PTGDR
IL10RA
IL33
MEFV
GSDMC
CXCR4
CDH5
CYP26B1
DUOX2
RAB44
SLAMF8
TXK
PIK3AP1
P2RX7
FZD4
TLR3
IFITM2
PLA2G4C
BIRC3
PTGER2
NR4A1
IL18R1
FOS
PLCG2
TNIP3
LRFN5
CX3CL1
CXCL1
OAS1
APOL3
TSLP
IRF5
PTPN6
C4A
IL1RL2
AIM2
TLR2
IER3
IL16
FOXC1
IRGM
GPR68
CCN4
STAT4
FGR
MAPT
GATA3
IL34
GPRC5B
CCN3
C4B
IFNLR1
IL27
CSRP3
ITGAL
IL1RN
EREG
IL23A
CASP1
CCL26
IFI27
IL1R2
NR5A2
LGALS9
IL22RA1
TNFAIP6
EXT1
CD44
VCAM1
EDN1
CXCL8
CIITA
PTGS2
KLF4
IL6
USP18
CCL2
TRAF1
ISG15
NFKBIA
EGR1
TRIM14
IL15
PTN
CSF1
IL7R
JUN
LYN
PARP9
NAMPT
PIK3R1
IRAK2
RIPK2
PTK2B
NOD1
MAP3K8
PRLR
NMI
IL1A
IRF7
IFI35
ANGPT1
IL15RA
TRIM21
THEMIS2
ZFP36
S1PR3
ZC3H12A
RELB
IKBKE
ADM
SOCS3
IL7
TICAM1
ASS1
PRKCD
CASP4
LOXL3
CD70
RHBDF2
CASP8
RBM47
MYD88
BCL6
NINJ1
FAS
RPS6KA5
DUSP10
CREBRF
SLC39A8
SH2B3
HLA-E
IL1B
STAT5A
TRAF3IP2
IL12RB2
MMP12
METRNL
CD36
IL1RAPL2
ABCD1
DOK1
CEBPB
ADCY7
IL11
DAB2IP
ANXA4
CYLD
VRK2
ADAM17
NOTCH1
BMPR1B
TRIM56
TXNIP
MFHAS1
HIF1A
OTUD4
ELF4
SNX6
RELA
PSEN1
CAMK4
NEK7
PRKD1
ROBO1
STAT1
NLRC5
IFI16
EIF2AK2
RICTOR
EZH2
DICER1
IL6ST
ABHD12
RIPK1
CD47
NFKB1
IL1R1
ACVR1
LIPA
HDAC9
PLSCR1
SAMHD1
SP100
STAT2
NR3C1
ZEB2
FYN
PLCB1
TNFAIP3
OASL
TNIP1
JAK2
MX1
PARP14
SLIT2
IFIH1
APOL2
NFKBIZ
0.75
0.50
0.25
0.00
-0.25
-0.50
-0.75
z-score

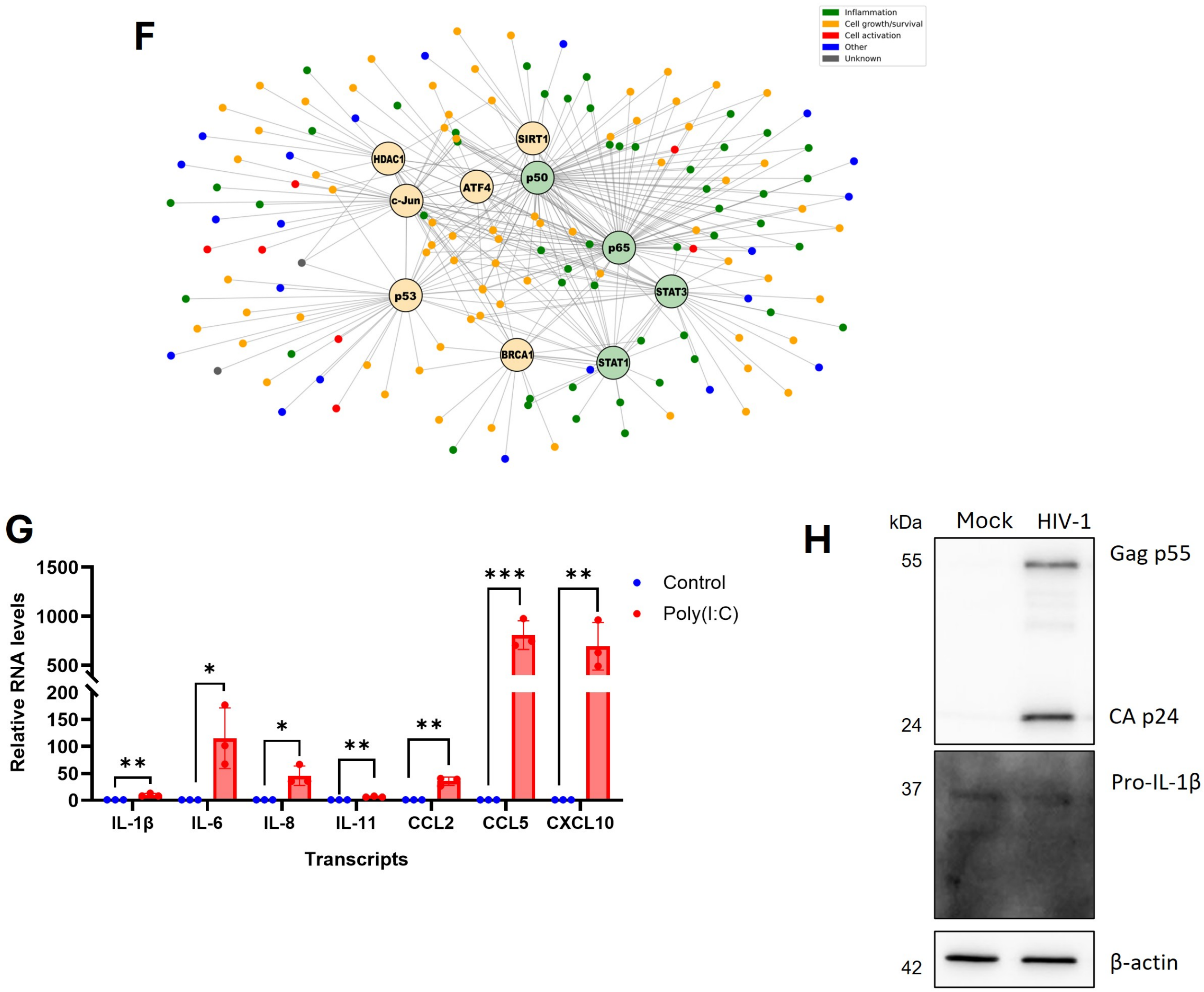

Supplementary Figure 2. NF-κB regulation during HIV-1 infection and poly(I:C) transfection in human microglia.
(A) Analysis of NF-κB subunits localization by subcellular fractionation (Cyto, Cytoplasm; Nuc, Nucleus) and representative western blot analysis (left and middle panel). Left panel: Fractionation of HIV-1-infected microglias at 0, 1 and 2 days post-infection. Middle panel: Subcellular fractionation of untransfected and poly(I:C) transfected cells. Antibodies against NF-κB family members, including p65, p105/p50, as well as HIV-p24 were used. *β*-tubulin and Lamin A antibodies served as cytoplasmic and nuclear fractionation controls, respectively. Immunofluorescence confocal microscopy of NF-κB p65 localization (right panel) in uninfected cells, HIV-1 infected cells for 2 days and poly(I:C) transfected cells. NF-κB p65 is shown in red, HIV-p17 in green and nuclei were stained with DAPI in blue. Scale bar: 20 µm. **(B)** C20 cells infected for 1, 3, 6 and 12 hours were subjected to immunofluorescence against NF-κB p65 (red). Images were obtained with an inverted epifluorescence microscope. **(C)** RT-qPCR analysis of HIV-1 viral transcripts (US: unspliced, PS: partially spliced, CS: completely spliced) in HIV-1-infected microglia treated with the NF-κB inhibitor parthenolide (10 µM). Viral transcripts levels were normalized to infected vehicle-treated control cells (DMSO). Data represents mean ± SD of five biological replicates. **(D)** RelB luciferase reporter activity in microglia infected with HIV-1 for 2 days. Luciferase activity was measured at 2 days post-infection and normalized to uninfected reporter cells. Data represent mean ± SD of five biological replicates. **(E)** Transcriptional regulatory network analysis showing the top 10 transcription factors associated with

poly(I:C)-induced upregulated genes. Nodes represent transcription factors and circles represent target genes. Elements are colored according to cellular processes: Green (Inflammation), Yellow (Cell growth/survival), Red (Cell activation), Blue (Other) and Grey (Unknown). **(F)** Hierarchical clustering of genes associated to inflammation annotated within the "inflammatory response" (GO:0006954) and "cytokine-mediated signaling pathway" (GO:0019221) obtained from RNA-sequencing data from control and cells transfected with poly(I:C). Up-regulated genes are indicated in the heatmap. **(G)** RT-qPCR analyses of IL-1β, IL-6, IL-8, IL-11, CCL2, CCL5 and CXCL10 relative mRNA levels in cells transfected with poly(I:C). Data is presented as mean ± SD of three biological replicates. Statistical significance is indicated as: ns, not significant; *, $p<0.05$;**, $p < 0.01$; ***, $p < 0.001$; ****, $p < 0.0001$. **(H)** Cells infected for 2 days were subjected to western blot against IL-1β. The unprocessed pro-IL-1β is indicated. Images are representative of three independent experiments.

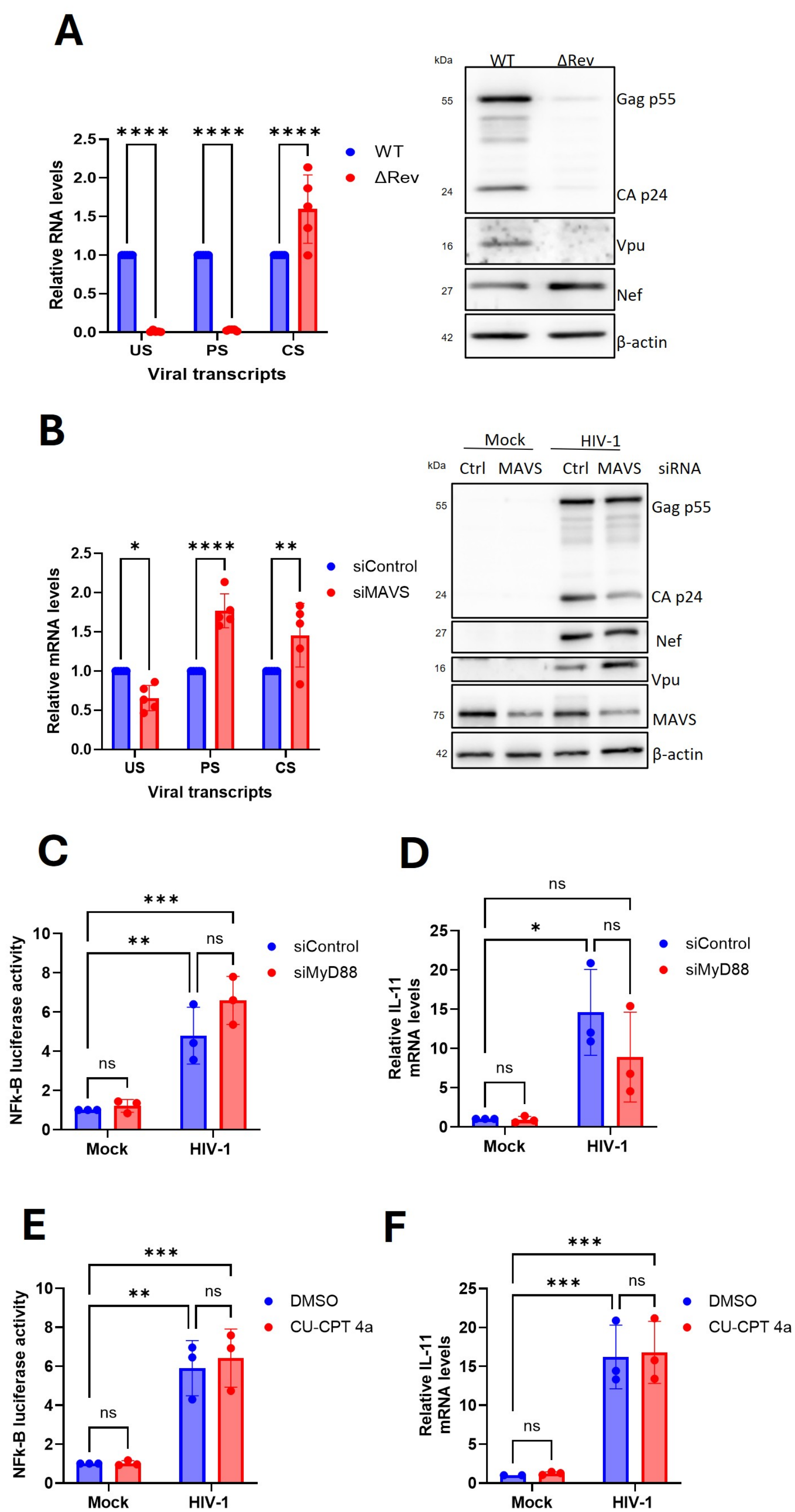
A
kDa
WT
ΔRev
55
Gag p55
24
CA p24
16
Vpu
27
Nef
42
β-actin
Relative RNA levels
****
WT
ΔRev
US
PS
CS
Viral transcripts
B
Mock
HIV-1
Ctrl
MAVS
siRNA
Gag p55
CA p24
Nef
Vpu
75
MAVS
β-actin
Relative mRNA levels
siControl
siMAVS
C
NFk-B luciferase activity
***
**
ns
siControl
siMyD88
Mock
HIV-1
D
Relative IL-11 mRNA levels
E
DMSO
CU-CPT 4a
F

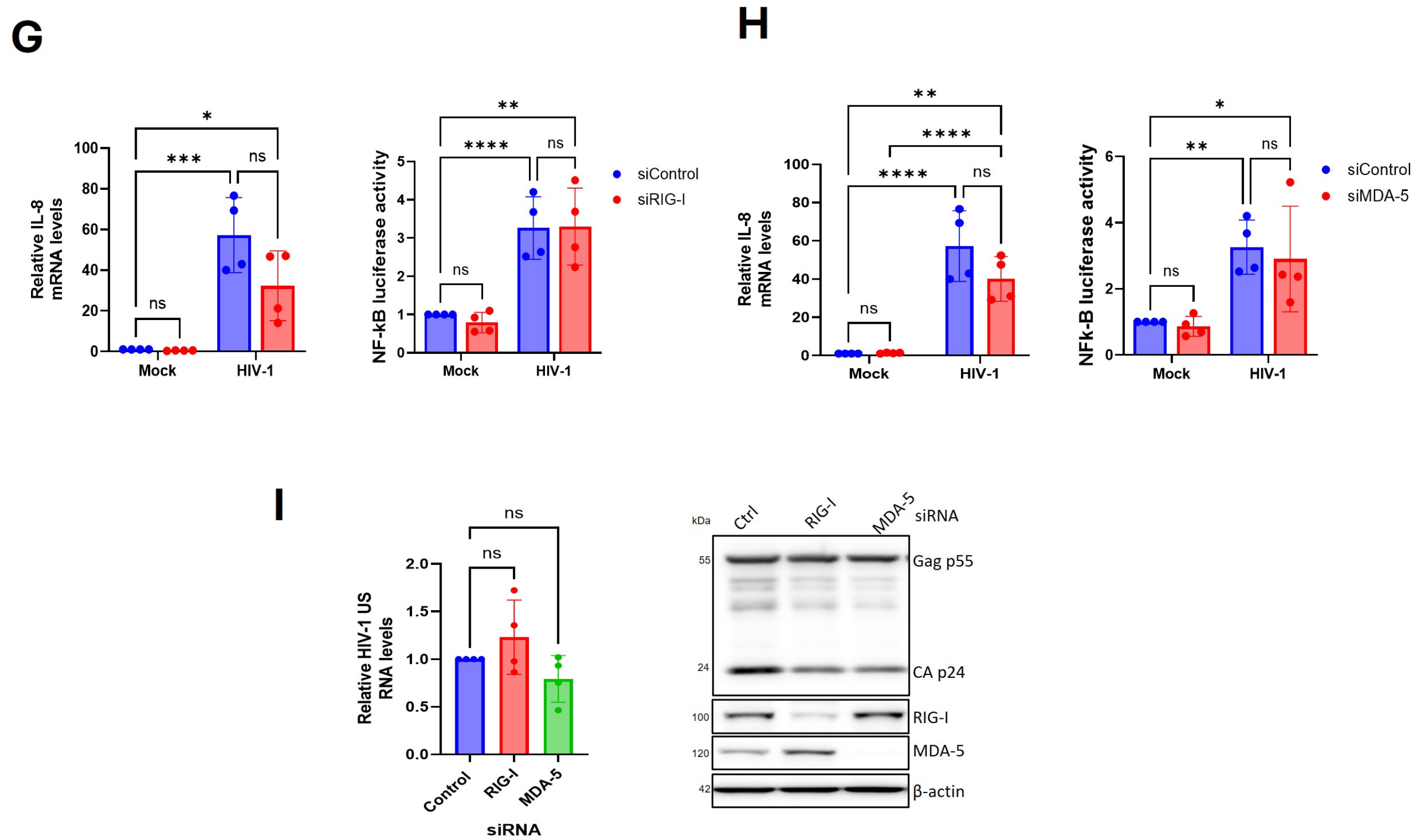

Supplementary Figure 3. Expression of Rev-dependent transcripts triggers IL-11 induction in a MAVS signaling-dependent manner.

(A) RT-qPCR of HIV-1 viral transcripts (US: unspliced, PS: partially spliced, CS: completely spliced) (left) and western blot analysis of viral proteins expression in cells infected with equal RNA copy numbers of Wild-Type (WT) HIV-1 or a replication-deficient mutant lacking Rev expression (ΔRev) (right). Antibodies against HIV-1 p24, Vpu and Nef were used. β-actin served as loading control. Data is representative of n=five biological replicates. **(B)** Cells were transfected with non-targeting control siRNA (siControl) or siRNA targeting MAVS (siMAVS) for 24 hours prior infection with HIV-1 for 2 days. Expression of HIV-1 viral transcripts (left) and proteins (right) were evaluated by RT-qPCR and western blot, respectively. Data is representative of n=five biological replicates.

(C,D) Cells were transfected with non-targeting siRNA control (siControl) or siRNA targeting MyD88 for 24 hours prior to infection. Relative NF-kB luciferase activity **(C)** and levels of IL-11 mRNA (D) were determined at 2 days post-infection. **(D,F)** Cells were treated with the TLR3 inhibitor CPU-CPT 4a for 24 hours prior to infection. Fresh media containing additional inhibitor was added at 24 hours post-infection during media change. Relative NF-kB luciferase activity **(D)** and levels of IL-11 mRNA (F) were determined at 2 days post-infection. **(G,H)** Microglia and NF-kB microglia reporter cell lines were transfected with non-targeting control siRNA (siControl) or siRNA targeting RIG-I **(G)** or MDA-5 **(H)**. Relative IL-8 mRNA levels (left) as well as induction of the NF-kB reporter activity (right) was evaluated by RT-qPCR and luciferase activity, respectively. Data is representative of n=four biological replicates. **(I)** Cells were transfected with non-targeting control siRNA (siControl) or siRNA targeting RIG-I or MDA-5. Expression of HIV-1 unspliced (US) viral transcripts (left) and Gag protein (right) were evaluated by RT-qPCR and western blot, respectively. Data is representative of n=four biological replicates. Statistical significance is indicated as: ns, not significant; *, p<0.05;**, p < 0.01; ***, p < 0.001; ****, p < 0.0001.

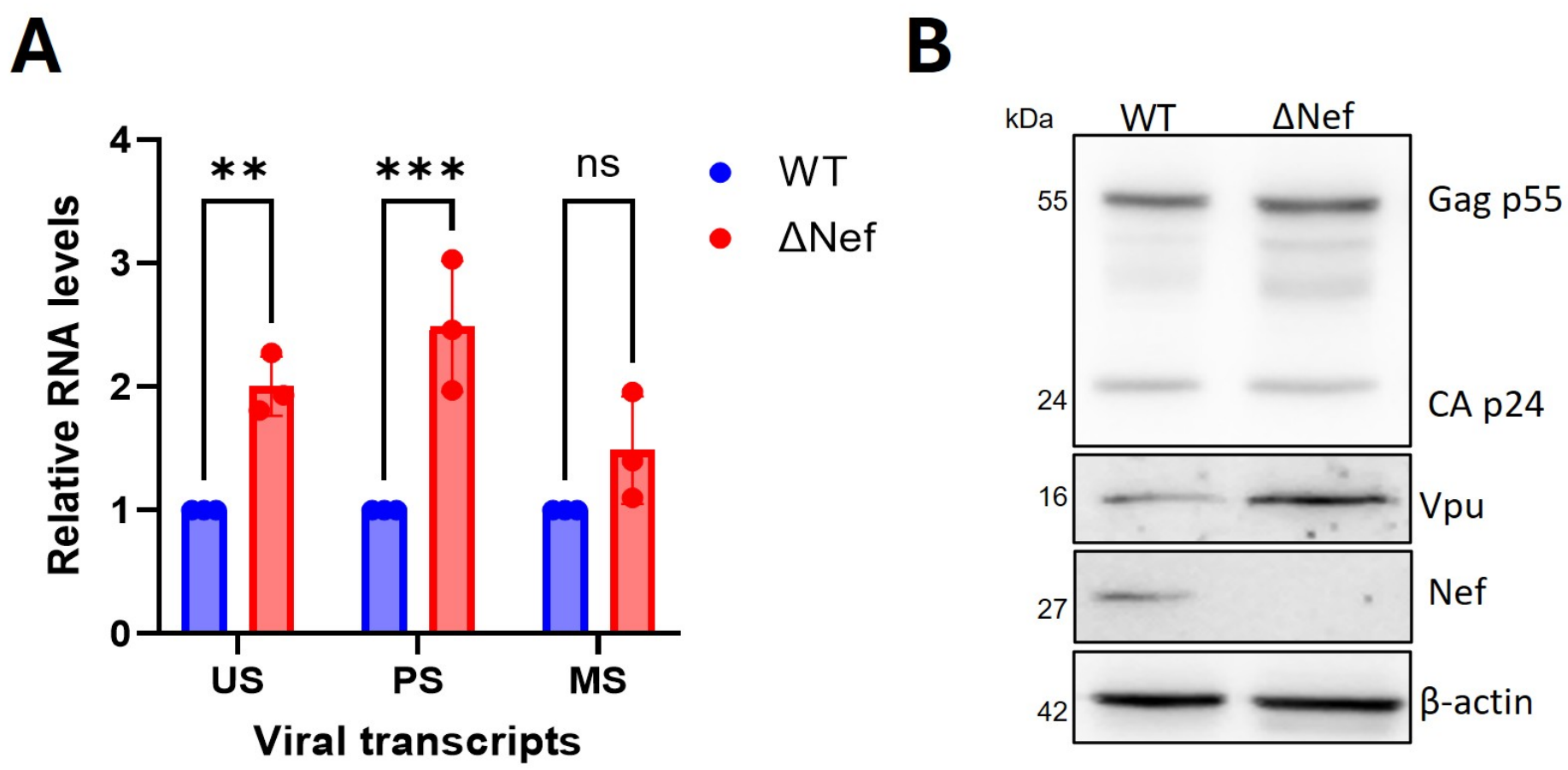

Supplementary Figure 4. HIV-1 replication is not affected by the absence of Nef.

(A,B) RT-qPCR of HIV-1 viral transcripts (US: unspliced, PS: partially spliced, CS: completely spliced) **(A)** and western blot analysis **(B)** of viral proteins expression in cells infected with equal RNA copy numbers of Wild-Type (WT) HIV-1 or a mutant lacking Nef expression (ΔNef). Antibodies against HIV-1 p24, Vpu and Nef were used. β-actin served as loading control. Data is representative of n=three biological replicates. Statistical significance is indicated as: ns, not significant; **, $p < 0.01$; ***, $p < 0.001$.
